# Fast Permeation of Small Ions in Carbon Nanotubes

**DOI:** 10.1002/advs.202001802

**Published:** 2020-12-20

**Authors:** Steven F. Buchsbaum, Melinda L. Jue, April M. Sawvel, Chiatai Chen, Eric R. Meshot, Sei Jin Park, Marissa Wood, Kuang Jen Wu, Camille L. Bilodeau, Fikret Aydin, Tuan Anh Pham, Edmond Y. Lau, Francesco Fornasiero

**Affiliations:** ^1^ Physical and Life Sciences Lawrence Livermore National Laboratory Livermore CA 94550 USA; ^2^ Howard P. Isermann Department of Chemical and Biological Engineering and Center for Biotechnology and Interdisciplinary Studies Rensselaer Polytechnic Institute Troy NY 12180 USA

**Keywords:** anomalous transport, carbon nanotubes, fast ion permeation, flow enhancement, nanofluidics

## Abstract

Simulations and experiments have revealed enormous transport rates through carbon nanotube (CNT) channels when a pressure gradient drives fluid flow, but comparatively little attention has been given to concentration‐driven transport despite its importance in many fields. Here, membranes are fabricated with a known number of single‐walled CNTs as fluid transport pathways to precisely quantify the diffusive flow through CNTs. Contrary to early experimental studies that assumed bulk or hindered diffusion, measurements in this work indicate that the permeability of small ions through single‐walled CNT channels is more than an order of magnitude higher than through the bulk. This flow enhancement scales with the ion free energy of transfer from bulk solutions to a nanoconfined, lower‐dielectric environment. Reported results suggest that CNT membranes can unlock dialysis processes with unprecedented efficiency.

## Graphical abstract

Concentration‐gradient driven diffusion rates of ions through single‐walled carbon nanotubes are shown to be up to 36 times faster than in bulk. This previously unrecognized flow enhancement scales with the ion free energy of transfer from bulk solutions to a nanoconfined, lower‐dielectric environment. Utilization of fast diffusion can greatly benefit fields ranging from hemodialysis to energy storage.

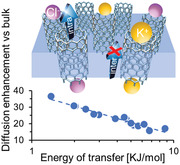

Molecular diffusion across a semi‐permeable membrane is crucial to many natural and man‐made processes.^[^ ^1^ ^]^ Biological membranes, such as those found in the kidney and liver, possess complex transport mechanisms at the nanoscale that enable both a high level of control over the profile of permeating molecules and fast filtration rates. Synthetic nanoporous materials aimed at reproducing these characteristics fail to deliver comparable permeability at the small pore diameters required for size‐sieving molecular separations. For example, membranes designed to reproduce kidney function are unable to provide sufficient throughput, causing patients to spend extensive time in the hospital.^[^ ^2^ ^]^

1D and 2D materials^[^ ^3^, ^4^ ^]^ have recently shown promise for enabling rapid and selective transport across membranes for blue‐energy harvesting,^[^ ^5^ ^]^ desalination,^[^ ^6^ ^]^ nanofiltration,^[^ ^7^ ^]^ and dialysis^[^ ^8^ ^]^ applications. Among these materials, carbon nanotubes (CNTs) offer an intriguing option because they emulate key features of many biological channels, both in their structure (small diameters, functionalizable tips, well‐defined interior)^[^ ^9^, ^10^ ^]^ and high transport efficiency^[^ ^11^ ^]^ enabled by their frictionless, hydrophobic surfaces.^[^ ^12^ ^]^ It is well established that certain modes of transport through the CNT lumen are dramatically enhanced above other pores of comparable size. Under a pressure driving force, flow enhancements range from one to two orders of magnitude for gases^[^ ^13^, ^14^, ^15^ ^]^ and three to five orders of magnitude for water^[^ ^13^, ^16^ ^]^ when compared with Knudsen and Hagen–Poiseuille models, respectively. Ion mobilities several orders of magnitude above bulk have been reported under a voltage gradient.^[^ ^17^ ^]^

In the case of a concentration driving force, however, the picture is less clear. The diffusion of small molecules/ions through CNTs is typically assumed to follow bulk or hindered diffusion models,^[^ ^18^, ^19^ ^]^ yet recent reports suggest that this may not be a valid assumption in all cases. Several simulations have indeed predicted enhanced self‐diffusivities for large ions inside CNT channels, and a few self‐diffusion experiments seem to support these claims.^[^ ^20^, ^21^, ^22^, ^23^, ^24^, ^25^, ^26^ ^]^ In addition, our previous reports have shown water vapor permeances under a relative humidity gradient that are 24–100 times larger than Knudsen theory estimates,^[^ ^14^, ^27^ ^]^ and others have measured proton diffusivity in very narrow CNTs exceeding bulk transport by ten times.^[^ ^28^ ^]^ Unfortunately, conclusive experimental validation of enhanced diffusion in liquid phases has remained elusive thus far.^[^ ^29^ ^]^ This gap persists due to challenges in fabricating defect‐free membranes with precisely characterized number and size of transport channels, and the presence of boundary layer resistances that may dominate and obscure transport behavior.

In this work, we show that the concentration‐driven permeation of small ions through single‐walled carbon nanotubes (SWCNTs) exceeds transport through the bulk by more than one order of magnitude. This fast diffusion is surprisingly displayed for ion/pore size ratios that would typically result in hindered diffusion.^[^ ^18^ ^]^ These discoveries were enabled by the use of membranes containing vertically‐aligned SWCNTs (VA‐SWCNT) with narrowly distributed diameters as the only transporting pathways. We performed rigorous and novel control experiments to confirm that our membranes contain no detectable defects with sizes either below or above the CNT pore diameters. Fine tuning of the number of transporting CNTs in each membrane also enabled correction for the large contribution of the boundary layer resistance at the membrane surface. Harnessing such large diffusion rates of small‐ions/molecules in SWCNT devices has the potential to benefit many applications such as hemodialysis,^[^ ^30^ ^]^ peptide/protein/bioconjugate purification and recovery,^[^ ^31^ ^]^ algae cultivation/harvesting,^[^ ^32^ ^]^ and energy storage.^[^ ^33^ ^]^

To accurately quantify the ion diffusion rate through a‐few‐nm wide CNTs (average diameter of 2.2 nm, Figure S2, Supporting Information), we fabricated free‐standing membranes by infiltrating high quality SWCNT forests (having a known number of nanotubes) with parylene (**Figure** **1**A),^[^ ^14^ ^]^ and performed a suite of stringent tests to rule out leaky transport pathways (i.e., other than CNTs). During membrane opening via plasma etching, we observed a rapid increase in the gas permeance followed by a plateau with increasing etch depth (Figure 1B), which was estimated from the etch rate of parylene coated on Si chips (see Materials and Methods, Supporting Information). This etch curve indicates both a complete opening of available CNT channels and a high quality of parylene infiltration, as the permeance through defects in the parylene matrix would be expected to increase with additional etching. As further evidence of transport through nanometer‐wide channels and negligible viscous flow through large matrix gaps, the N_2_ permeance in these membranes was independent of pressure. These membranes were also subjected to rejection tests and only those blocking >99.5% of a probe dye (Direct Blue 71, 1.5 × 1 × 3 nm) were employed for following diffusion studies.

**Figure 1:**
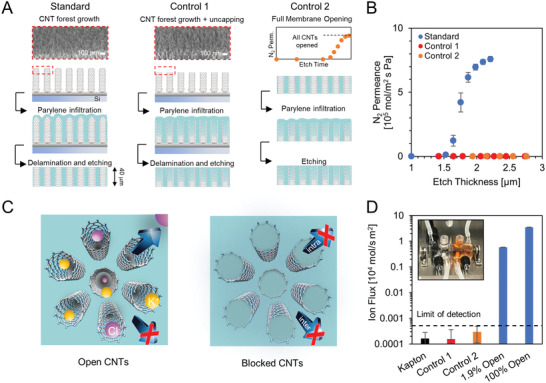
Absence of leakage pathways in fully opened CNT membranes. A) Fabrication steps for a standard membrane (left column), C1 (middle), and C2 (right) controls. Scanning electron microscopy images show the forest top layer following growth (left) and plus a 5 min air plasma etch (middle). The graph in the right column shows a representative opening curve for the membranes used in C2 control fabrication. B) Nitrogen permeance plotted as a function of etching time for a standard membrane (blue) and controls (C1 red and C2 orange). Error bars represent the standard deviation for three membranes. C) Schematic representation of the standard (left) and control (right) membranes with open and blocked CNTs, respectively. D) KCl flux measured under a 50 mm concentration gradient at 25 °C through control membranes (orange and red), a solid piece of Kapton polyimide (black), and standard CNT membranes with 1.9% and 100% of the CNTs opened (blue). The limit of detection is defined as the flux through the polyimide plus 3 × the standard error (dashed line). Error bars represent the standard error from the linear fit of permeate conductivity versus time. Inset: Schematic of the diffusion set up (Figure S4, Supporting Information).

While these quality tests are comparable with those currently accepted in the CNT membrane literature (see Table S1, Supporting Information), they do not conclusively rule out alternative transport pathways with diameters that are equal to or smaller than the CNT pores. To demonstrate that CNTs are the only fluid conduits, we intentionally blocked transport through their interiors with parylene. The absence of fast flow and high dye rejection in these clogged‐CNT controls would provide exceptionally strong evidence that recorded transport in our standard membranes is indeed only through CNT channels. To this goal, we fabricated two types of clogged‐CNT control membranes (Figure 1A,C). In the first design (C1), CNT forests were exposed to a low‐power air‐plasma etch for 5 min to remove the native CNT caps prior to parylene infiltration (Figure 1A and Figure S5, Supporting Information). In the second design (C2), a fully‐opened standard membrane was subjected to a second round of both parylene infiltration and plasma etching. Parylene‐N was selected for the clogging step in both controls due to its ability to better plug the CNT interiors.^[^ ^34^ ^]^ Since the ion diffusion dataset reported below employed membranes with both parylene‐C and parylene‐N matrices, we selected fully‐opened parylene‐C membranes for C2. Datasets separated by parylene type show excellent agreement in ion diffusion enhancement (Figure S3, Supporting Information).

For these controls, the extensive etch step after CNT clogging is expected to either form defects (C1 and C2) or uncover those already present (C2). Five sample membranes of each control type were etched to depths of at least 1.24 × those sufficient to complete CNT opening in a standard membrane (1.79 × the depth to initiate opening), and the majority showed no N_2_ transport (Figure 1B). Etching beyond this depth resulted in a high rate of large‐scale membrane failure, preventing further transport measurements. The small subset of control membranes that showed some N_2_ permeance dramatically failed the dye test. For membranes with zero N_2_ permeance, KCl diffusive flux was also found to be at or below the detection limit of our conductivity probe and comparable to that of a solid Kapton film (Figure 1D). Thus, leaky transport pathways formed in clogged‐CNT membranes cannot be responsible for the high transport rates and selectivity recorded with open SWCNT conduits. These control experiments prove that, in our diffusion studies with standard open membranes, fluid flow occurs only through SWCNT pores.

Diffusive transport through our membranes was quantified by recording the salt concentration change in the receiving chamber of a diffusion cell as a function of time (see Materials and Methods, Supporting Information). Unless otherwise noted, all experiments were conducted at pH = 3 using 50 mm and no salt in the high and low concentration reservoirs, respectively. Solution pH had negligible impact on results (Figure S9, Supporting Information). Experimentally measured fluxes include the contribution of both the membrane (*R*_mem_) and boundary layer resistances (*R*_BL_) on either side of the membrane. As a result, we define here an effective enhancement factor *EF* * as the ratio of the measured flux, *F*_meas._, to that expected assuming bulk diffusion in the membrane pores, *F*_bulk_. To characterize diffusive transport in our open membranes alone, which depends both on the solute concentration in the membrane and its diffusion coefficient, we define also an enhancement factor, EF, as the ratio of the permeability constant within a membrane, *K*_H2O − mem_  ×  *D*_mem_, to *D*_bulk_. Here, *K*_H2O − mem_ represents the partition coefficient between bulk solution and the membrane, while *D*_mem_ and *D*_bulk_ are the diffusion coefficients in the membrane and bulk, respectively. If each boundary layer has thickness *δ*, we can then relate *EF* and EF* with

(1) $$\begin{array}{c} \frac{1}{EF^{*}}\;=\;\frac{1}{EF}+2\varphi \frac{\delta }{\tau L_{\mathrm{mem}}} \end{array}$$

where *φ*, *L*_mem_ and *τ* are the porosity, thickness, and tortuosity of the membrane, respectively. Using this relationship, both *R*_BL_ and *EF* can be extracted from a set of diffusion experiments through membranes with different transport resistances (Section S7, Supporting Information).

To validate our platform and experimental procedure, we first used a set of commercially available polycarbonate track‐etched (PCTE) membranes (pore diameters from 34 to 415 nm, see Table S3, Supporting Information), and fitted 1/EF* versus 1/*R*_mem_ data for five different salts, spanning a range of diameters and valences. The results corroborate the expected transport behavior (Figure S6, Supporting Information):^[^ ^35^ ^]^ *EF* ≈1 confirms bulk transport in the large PCTE pores for all salts, and *R*_BL_ shows good agreement with the theoretical $R_{\mathrm{BL}}\;\approx \;D_{\mathrm{bulk}}^{2/3}\;$scaling with the bulk salt diffusion coefficient.

Similar experiments were carried out using a set of CNT membranes fabricated from a single chemical vapor deposition growth with a controlled number of open channels, from 1.9% to 100% of the SWCNTs in the membrane (1.67 × 10^12^ cm^−2^). Opening percentage was calculated by comparing the membrane nitrogen permeance with the average permeance of three fully opened membranes from the same batch. Values for *K*_H2O − mem_  ×  *D*_mem_ significantly exceed bulk transport (Table S4, Supporting Information), with *EF* ranging from 17 to 36 (**Figure** **2**A,B), whereas *R*_BL_ was found to match with that observed for PCTE (Figure S6B, Supporting Information). Measured boundary layer resistance is an order of magnitude larger than that of a fully opened CNT membrane and thus dominates the overall resistance. This provides a possible explanation for the lack of experimental evidence of faster‐than‐bulk diffusive transport for ions in SWCNTs until now (Figure 2C). The recorded magnitude of diffusion enhancement lends further evidence that bulk diffusion through leaky pathways cannot explain the observed transport rates: for several salts, a hypothetical bulk diffusion through the entire membrane area would not be sufficient to supply the measured ion flux through a fully opened CNT membrane (≈6.4% CNT porosity).

**Figure 2:**
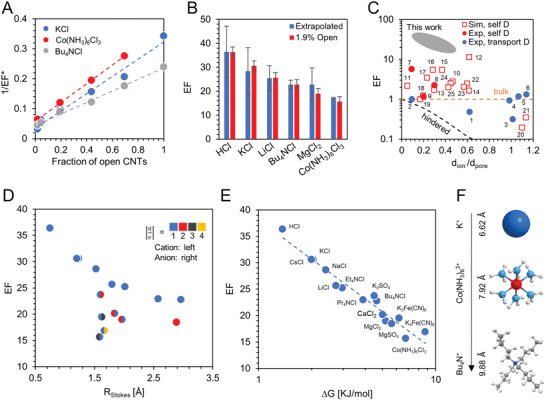
Fast ion diffusion through CNTs. A) 1/EF* versus the percentage of open CNTs for KCl (blue), Bu_4_NCl (grey), and Co(NH_3_)_6_Cl_3_ (red). Dashed lines represent a linear fit with Equation (1). Error bars are calculated from the standard deviation of the maximum N_2_ permeance for the three fully opened membranes (*x* direction) and from three repeated diffusion experiments with the same membrane (*y* direction) and are smaller than the data points. B) Comparison of *EF* obtained by extrapolation from the entire dataset of membranes with varying degree of opening (blue) and from a single 1.9% open membrane (red). Error bars come from the quality of the linear fit to 1/EF* versus percentage of CNTs open (extrapolated dataset, dashed line in panel A) and from three repeated experiments using the same membrane (1.9% open dataset). C) Partial summary of published literature indicating that ion diffusion may be enhanced under graphitic nanoconfinement (CNTs, graphene slits, or pores in activated carbon; see Table S2, Supporting Information). Hydrated ion size is used for data collected in aqueous environments, and *d*_pore_ is taken as the smallest nanochannel dimension. Red squares and circles are self‐diffusion data from simulations and experiments, respectively; blue circles are transport diffusion from experiments which assumed *K*_H2O − mem_ equal to 1; orange dashed line is bulk diffusion; black dashed line is predictions from hindered diffusion model; gray oval shows results from this work. D) *EF* plotted versus the Stokes radius for the salt, *R*_Stokes_ =  (*r*_cat_ *ν*_cat_ + *r*_an_ *ν*_an_)/(*ν*_cat_ + *ν*_an_). Coloring of the left and right datapoint halves represent the absolute value of the anion and cation charge number z, respectively (blue = 1, red = 2, dark gray = 3, orange = 4). E) *EF* plotted as a function of the energy penalty calculated for a charged hard sphere moving from bulk water to water confined in a CNT (Equations (2) and (3)). In (D) and (E) all data points are taken from a single 1.9% open membrane for which *R*_BL_ is negligible. F) Molecular model of three cations tested with their corresponding hydrated diameter and charge.

To better understand the nature of this enhanced transport we selected our highest‐resistance membrane for an extended salt sweep. Fifteen different salts were tested with varying size and charge of the constituent anions and cations. At an opening of only 1.9% the boundary layer resistance is negligible as revealed by the close match between *EF* and *EF* * (Figure 2B). Measured *EF* does not show a clear trend with either ion size or charge alone (Figure 2D). *EF* does scale, however, with the free‐energy penalty an ion must pay to move from bulk solution to the confined CNT interior, Δ*G*, as shown in Figure 2E. Here Δ*G* was estimated as the sum of the energy required to confine a neutral hard sphere in a narrow cylinder, Δ*G*_HS_, and the electrostatic energy to transition ions from bulk water to a solution of reduced dielectric constant (the pore interior), Δ*G*_el,H2O − CNT_ (Section S15, Supporting Information).^[^ ^36^ ^]^ We calculated Δ*G*_HS_ as^[^ ^18^ ^]^

(2) $$\begin{array}{c} \Delta G_{\mathrm{HS}}=-RTln\left[{\left(1-\frac{r_{\mathrm{cat}}}{r_{\mathrm{CNT}}}\right)}^{2\nu_{\mathrm{cat}}}{\left(1-\frac{r_{\mathrm{an}}}{r_{\mathrm{CNT}}}\right)}^{2\nu_{\mathrm{an}}}\right] \end{array}$$

with *ν* defined as the stoichiometric coefficient, *R* the gas constant, *T* the temperature, *r*_CNT_ the average inner CNT radius, and *r*_cat_ and *r*_an_ the Stokes radius of the cation and anion, respectively. Δ*G*_el, H2O − CNT_ was estimated with

(3) $$\begin{array}{c} \Delta G_{\mathrm{el},\;H2O-\mathrm{CNT}}=\frac{N_{A}e^{2}}{4\pi \varepsilon_{0}}\;\left(\frac{\nu_{\mathrm{cat}}z_{\mathrm{cat}}^{2}}{r_{\mathrm{cat},\;\mathrm{eff}}}+\frac{\nu_{\mathrm{an}}z_{\mathrm{an}}^{2}}{r_{\mathrm{an},\;\mathrm{eff}}}\right)\left(\varepsilon_{\mathrm{CNT}}^{-1}-\varepsilon_{H2O}^{-1}\right) \end{array}$$

where *N*_A_ is Avogadro's number, *z* is the valence, *ε*_0_ is the permittivity of vacuum, *ε*_H2O_ and *ε*_CNT_ are the relative permittivity of bulk water and water inside a CNT, respectively, and *r*_eff_ is the effective ion radius, as defined by Marcus.^[^ ^36^ ^]^ In Figure 2E, *ε*_CNT_ was fit to the experimental data while keeping all other parameters fixed. Fit quality was high (*R* ^2^ = 0.95) indicating an exponential relationship with Δ*G* despite significant variation in ion charge and size. A comparison with the potential of mean force calculations for a subset of ions (Section S16, Supporting Information) shows that Equations (2) and (3) capture correctly both the order of magnitude and the free‐energy penalty trend with ion type. Fitted *ε*_CNT_ = 75.4 is lower than that of bulk water, in agreement with literature reports that suggest reorientation of water dipoles along the nanotube axis reduces the radial component (and resulting “average”) of the dielectric constant of nanoconfined water.^[^ ^37^ ^]^

Several hypotheses were considered as possible explanations for this enhanced diffusive transport. First, we speculated a large contribution arising from diffusion‐osmotic bulk fluid motion, which was recently reported in CNTs.^[^ ^38^ ^]^ Measured water flux through our membranes, however, was found to be towards the high concentration reservoir, that is, in the opposite direction with respect to the ion flux. Also, the water flow rate was only 0.05–0.6 fL h^−1^ per CNT, depending on the salt solution, which was a few orders of magnitude lower than needed to explain our enhanced transport (Section S10, Figure S7, Supporting Information). Furthermore, we quantified the Zeta potential across our membranes using streaming current recording under a pressure gradient. For salts with unequal ion diffusion coefficients, electro‐osmosis may indeed induce an additional contribution to the diffusion–osmotic flow with magnitude proportional to the Zeta potential.^[^ ^1^ ^]^ Regardless of the slip length used in the calculation, the measured Zeta potential (−2.4 ± 1.2 mV in a 50 mm KCl solution at pH 3) was far below the level required to quantitatively match our diffusion rates (Figure S8, Supporting Information).^[^ ^1^ ^]^ These experiments conclusively exclude diffusion–osmosis as the origin of the reported transport phenomena.

Second, we considered that the enhanced ion flux could come from an increased self‐diffusion coefficient (*D*_self_) of confined ions and water. For example, this could arise due to a reduced density and a disrupted electrostatic^[^ ^22^ ^]^ or hydrogen bond^[^ ^39^ ^]^ network inside a CNT. To test this hypothesis, we performed pulsed field gradient stimulated‐echo nuclear magnetic resonance (NMR) measurements on membranes with CNT pores filled by a LiCl aqueous solution (**Figure** **3**C and S10, Supporting Information). Self‐diffusivities of Li^+^ ions and water inside the CNT were only slightly elevated (1.2 × 10^−9^ and 2.2 × 10^−9^ m^2^ s^−1^, respectively) compared to bulk (1.1 × 10^−9^ and 2.0 × 10^−9^ m^2^ s^−1^). Complementary classical simulations with a polarizable force field in 1.5 nm SWCNTs (Figure 3 and S11, Supporting Information) also recovered self‐diffusion coefficients and hydration numbers that were near bulk values. Thus, a significant ion *D*_self_ increase in our system appears to be unlikely, and the small *D*_self_ enhancement we measured by NMR cannot quantitatively explain our LiCl fluxes.

**Figure 3:**
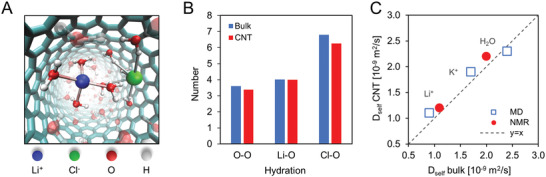
Computational predictions and NMR analysis of ion self‐diffusion. A) Molecular dynamics (MD) snapshot showing Li^+^ (blue), Cl^−^ (green) ions, and their solvation shells (indicated by visible water molecules) inside of a 1.5 nm diameter CNT filled with water. B) Hydration numbers of the first solvent shell for water, Li^+^ and Cl^−^ from MD simulations in bulk (blue) and inside a CNT (red). The first minima in the radial distribution function (RDF) between the ion and water oxygens were used as the distance cutoff for determination of the hydration number of the first ion solvation shell, which is 2.7 and 3.8 Å for Li^+^ and Cl^−^, respectively. C) Comparison of the self‐diffusion found from NMR (Li^+^, H_2_O) and MD simulations (Li^+^, K^+^, H_2_O) in bulk and under CNT confinement. Plots of mean square displacement (MSD) for self‐diffusion coefficient determination, RDF, and ion coordination number are shown in Figure S11, Supporting Information.

Other theoretical studies have reported ion transport diffusivities inside neutral graphitic channels surpassing bulk diffusion by 2‐4 fold,^[^ ^24^ ^]^ which was explained by an incomplete ion solvation and lower average density of water molecules under confinement.^[^ ^25^ ^]^ Finally, a recent computational model suggested that phonon‐induced oscillations in the friction force between water and CNTs^[^ ^40^ ^]^ may result in transport diffusivity a few times larger than in the bulk. To our knowledge, these single‐digit enhancement values are the closest results to our experimental data reported in the literature, yet they are still comparatively low.

In summary, we demonstrated permeabilities of small ions in SWCNTs that are more than one order of magnitude larger than in the bulk. This previously unreported fast diffusion occurs in a regime where bulk or hindered transport would typically apply and scales with the ion free energy of transfer from bulk to nanoconfined water. Several possible mechanistic explanations of the observed fast flow were considered, but none of the proposed phenomena can quantitatively capture the magnitude of diffusive flow enhancement observed in this study. The quantitative mismatch between experimental results and expectations highlights a need for further, independent investigations toward elucidating the mechanism underlying the observed fast ion diffusion. This unexpected transport behavior expands the number of unusual and often poorly understood nanofluidic phenomena recently observed in single‐digit nanopores.^[^ ^4^, ^41^ ^]^ Regardless, our discovery opens opportunities to exploit CNT‐based devices to achieve unprecedented performance metrics in several application areas, from dialysis (Figure S13, Supporting Information) and bioseparation processes to drug delivery and energy storage.

## Conflict of Interest

The authors declare no conflict of interest.

## Author Contributions

S.F.B., M.L.J., and C.C. fabricated the SWCNT membranes. S.J.P and E.R.M. synthesized and characterized the SWCNT forests. M.L.J. and S.F.B. performed pressure‐driven liquid and gas transport measurements. S.F.B. and M.W. performed diffusion and streaming current measurements. A.S. obtained and analyzed the NMR self‐diffusivity data. T.A.P., E.Y.L., F.A., and C.L.B. carried out molecular dynamics simulations. F.F. and S.F.B. interpreted the experimental data. F.F. planned activities, guided the experiments, and coordinated the paper preparation. All authors contributed to manuscript writing.

## Supporting information

Supporting Information
