# Supplementary material for: Fast Permeation of Small Ions in Carbon Nanotubes
Source: Adv Sci (Weinh). 2020 Dec 20;8(3):2001802. doi: 10.1002/advs.202001802 (PMC7856893; doi:10.1002/advs.202001802)
Supplement: Supplementary file 1 — Supporting Information [file ADVS-8-2001802-s001.pdf]

© 2020 Wiley-VCH GmbH

ADVANCED SCIENCE
Open Access

Supporting Information

for *Adv. Sci.,* DOI: 10.1002/advs.202001802

Fast Permeation of Small Ions in Carbon Nanotubes

*Steven F. Buchsbaum, Melinda L. Jue, April M. Sawvel, Chiatai Chen, Eric R. Meshot, Sei Jin Park, Marissa Wood, Kuang Jen Wu, Camille L. Bilodeau, Fikret Aydin, Tuan Anh Pham, Edmond Y. Lau, Francesco Fornasiero**

Supporting Information

Fast Permeation of Small Ions in Carbon Nanotubes

*Steven F. Buchsbaum, Melinda L. Jue, April M. Sawvel, Chiatai Chen, Eric R. Meshot, Sei Jin Park, Marissa Wood, Kuang Jen Wu, Camille L. Bilodeau, Fikret Aydin, Tuan Anh Pham, Edmond Y. Lau, Francesco Fornasiero**

*Email: fornasiero1@llnl.gov

This file includes:

Materials and Methods

Supplementary Text

Figure S1 to S13

Table S1 to S4

Materials and Methods

SWCNT growth: Carbon nanotube forests were synthesized from $Fe/Mo/Al_2O_3$ multilayer, thin-film catalysts following our previously reported procedure.[1] Each layer was sequentially deposited onto 100 mm Si (100) wafers by electron-beam evaporation without breaking vacuum (base pressure $\leq 1.6 \times 10^{-6}$ mbar). Nominal thicknesses of the catalyst layers used in this study ($Fe/Mo/Al_2O_3$ = 5.5/0.5/300 Å) were recorded *in situ* by a quartz crystal monitor during deposition. Synthesis of small diameter SWCNTs was performed at low pressure in a cold-wall furnace (AIXTRON® Black Magic), featuring a wafer-scale heater stage and gas showerhead. The chamber was pumped down below 0.2 mbar prior to initiating the growth recipe, which began with a thermal annealing step in a reducing environment before introducing the hydrocarbon feedstock growth gas (C_2H_2). For this study, we utilized the following recipe: ramp with top heater at 300 °C min^{-1} to 700 °C and bottom heater at 300 °C min^{-1} to 800 °C at 80 mbar in H_2/Ar = 700/200 sccm and holding for 2 min at 800 °C before switching the gas mixture to C_2H_2/H_2/Ar = 4/700/180 sccm at 80 mbar for 13 min (with addition of 20 sccm Ar through a bubbler containing H_2O, resulting in ~170 ppm$_v$). These low-pressure recipes produced forests with 30-50 μm thickness and number density > 10^{12} cm^{-2}.

SWCNT characterization: High-resolution transmission electron microscopy (HRTEM) was used to quantify the CNT diameter distribution and number of walls per CNT with a sample number $N \geq 65$ (image processing performed using custom MATLAB script, Figure S2B). We used a JEOL 2100-F field-emission analytical TEM, operating at 120 kV or 200 kV, ≥ 150 kx magnification, with a pixel resolution of ≤ 0.05 nm. We prepared samples by dispersing CNT forests in ethanol with ultrasonication and subsequently dropcasting the dispersion onto Cu TEM grids coated with

Formvar. Scanning electron microscopy (SEM) was performed with a Zeiss Gemini Ultra-55 analytical field emission SEM, operated at 5 kV accelerating voltage. The mean number density ρ_n (cm^{-2}) was quantified from the mass increase of the Si substrate after CNT growth[2] and from the forest height with the equation

$$\rho_{\mathrm{n}} = \frac{\rho_{\mathrm{m}}}{\pi d / SSA_{\mathrm{G}}} \tag{S1}$$

where ρ_m is the CNT volumetric mass density, d is the mean SWNT diameter measured by TEM, and SSA_G is the specific surface area of graphene, 1315 m^2 g^{-1}.

Micro-Raman spectroscopy of CNT forests was performed using a Renishaw InVia Qontor Raman spectrometer with excitation wavelength λ = 633 nm, a grating of 1200 lines/mm, and a 50x long working-distance objective. Final spectra used for analysis were generated by averaging over five 5-s collections. A representative spectrum from a forest used to produce CNT membranes in this work is reported in Figure S2.

Membrane fabrication: Standard CNT-parylene membranes were fabricated according to a previously reported process[3] with minor modifications. To form the CNT-parylene composite, the inter-tube spacing of the CNT arrays on Si wafers was infiltrated with parylene-C or parylene-N with a target coating thickness of 12000 Å (Specialty Coating Systems, Indianapolis, IN). Excess parylene was partially removed using an inductive-super-magnetron (ISM) generated oxygen plasma (ULVAC NE-550EXa). Antenna power was set to 200 W, bias power to 20 or 25 W, chiller to 0 deg C, pressure to 0.5 Pa and O_2 flow rate to 99 sccm. The etched composite wafers were then immersed in 37% hydrochloric acid (Sigma-Aldrich, product # 320331) for 5 to 24 hr to dissolve the alumina and catalyst layer between the CNTs and the Si-wafer and delaminate the CNT-parylene composite. The free-standing composites were cut into smaller

coupons and glued to 0.005-inch-thick Kapton-HN frames (Hardman Double/Bubble epoxy, product # 04007). Following N_2 permeance measurements to ensure all membranes were initially closed, alternating rounds of etching and transport measurements were performed until the desired permeance was reached. Etch steps were done using the same oxygen plasma method described above in steps of 25 s or less. Membranes were considered fully open when N_2 permeance varied by less than 3.5% between etching rounds. All membranes shown in Figure 2 were taken from a single 4-inch forest with an average diameter of 2.2 nm, height of 40 μm and CNT number density of 1.67 x 10^{12} cm^{-2}. We assumed a tortuosity of 1.25 in all calculations based on our previous results.[3] Two types of control membranes were also fabricated (C1 and C2). C1 membranes were exposed to a low power air plasma etch for 5 min (Harrick PDC-001, 30 W R_f) to remove the CNT caps prior to the parylene-N infiltration step (Figure S5). All further processing matched that of the standard membrane. C2 membranes were fabricated beginning with a fully opened standard membrane (parylene-C as matrix) which was subjected to a second round of parylene deposition. Additional rounds of etching and transport measurements were then performed in the same way as previously described.

<u>Plasma etch rate and depth determination:</u> A Si wafer was coated with parylene under the same conditions used during the membrane infiltration step and was later etched under conditions matching those used for membranes. Thickness of the parylene layer was measured at different points on the wafer after multiple etching steps using non-contact spectro-reflectometry (Nano Spec TOHO 3100). Data was fit to a Cauchy model with both thickness and index of refraction as fitting parameters. The etch rate was then determined by dividing the change in parylene

thickness by the time of sample etching. This etch rate was used to estimate the etch depth of CNT membranes for a given etch duration.

Gas transport measurements: N_2 permeance of SWCNT membranes was measured with a mass flow meter located downstream of a dead-end filtration cell, as previously reported.[3] Typically, the mass flow rate was recorded at 4 pressure points up to 2 psi. N_2 permeance measurements were used to gauge the degree of membrane pore opening (described in more detail below) and as a screening tool to qualitatively identify the presence of large defects. Since transport through a few nanometer wide SWNT pores is expected to be in the Knudsen regime and thus independent of the applied feed pressures, only membranes with N_2 permeance independent of pressure were used in this study.

Determining the number of transporting CNTs: Three representative membranes made from the same 4-inch forest as those reported in the main text were fully opened using the method described above. The averaged maximum N_2 permeance was then attributed to transport through all available CNT channels, the number of which is calculated by multiplying the known forest density by the membrane area. The number of transporting CNTs in partially opened membranes is then assumed to scale linearly with the N_2 permeance measured prior to downstream testing.

Water permeance and selectivity measurements: To characterize the selectivity of our membranes, we measured the rejection coefficient for a negatively charged dye, Direct Blue 71 (DB71, Sigma Aldrich, product # 212407), with size of 3×1.5×1 nm, as previously reported.[3] 10

μM DB71 aqueous feed solution was pressurized at 2 psi with a controlled in-house N_2 line in a dead-end filtration cell. Water permeance was quantified from the reduction of feed solution volume or from the increase of the permeate weight in a collection vial over time. After filtration, the solution permeated through the membrane was analyzed by UV-vis spectroscopy (Varian Cary 100 UV-Vis Spectrophotometer) to determine the analyte concentration and then calculate the rejection coefficient (R) using the equation:

$$R = 1 - \frac{A_P}{A_F} \tag{S2}$$

where A_P and A_F are the absorbance of the permeate and feed solution, respectively, at the peak wavelength λ=587 nm. Only membranes with a R above 99.5% were considered defect-free and used in this study. Due the possible presence of concentration polarization the reported rejection is a lower bound. Membranes fabricated following the same procedure as those in this work were also probed with neutral 5-nm diameter polyethylene-glycol-coated Au nanoparticles (0.05 mg/mL, NanoXact Gold by nanoComposix) and showed R > 99.5% (data not shown).

<u>Diffusion and water flux measurements:</u> Membranes were loaded into a custom designed Side-Bi-Side diffusion cell (Permegear, Figure S4) with integrated ports for a conductivity probe (SevenExcellence S700 with an INLAB 731-ISM probe, Mettler Toledo), glass capillaries, and temperature-controlled water jacket. The cell was kept on a magnetic stirring unit, and stir bars were placed on both sides of the membrane to keep solutions mixed. Custom silicone gaskets and O-rings were used to avoid leaks and seal the reservoirs, which enabled volume change measurements via the attached capillaries. Conductivity of the permeate reservoir, κ_p, was

recorded vs time, t, and the diffusion constant, *D*, of the permeating salt in the membrane was extracted by fitting the data to the following equation.

$$\kappa_p = \frac{\Lambda_m C}{2}\left(1 - e^{\frac{-2DNA\pi(\frac{d}{2})^2 t}{VL}}\right) + \kappa_{p,0} \quad \text{(S3)}$$

Here, Λ_m is the molar conductivity at infinite dilution, *C* is the initial concentration gradient across the membrane, *N* is the number density of transporting pores, *A* is the membrane area, *d* is the average pore diameter, *V* is the permeate reservoir volume, *L* is the membrane thickness and $\kappa_{p,0}$ is the permeate conductivity at the start of the experiment. The salt flux, F_{meas}, reported in Figure 1D of the main text was calculated assuming a constant *C* using the equation

$$F_{meas} = \frac{\Gamma V}{\Lambda_m A} \quad \text{(S4)}$$

where Γ is the slope of the linear fit to the permeate conductivity vs time. Water flux was measured via visually monitoring the height of the capillary (inner diameter = 0.375 mm) attached to each reservoir (Figure S7). Unless otherwise noted, 50 mM and no salt solutions were used for the initial feed and permeate concentrations, respectively, and pH = 3 was used in both permeate and feed solutions to minimize charge effects at the pore entrance/exit. (Dedicated tests at various pHs (Figure S9) have shown, however, that these effects are negligible in our experiments). For HCl diffusion measurements, a 50 mM concentration difference was maintained across the membrane, with the initial permeate solution set at pH 3. Tests on polycarbonate membranes were always done at pH 6 due to material incompatibility at low pH.

Streaming current measurements: Custom probes were fabricated using glass pipette capillaries and Ag/AgCl pellet electrodes (AM Systems). The conductivity probe was removed for these experiments, and the Ag/AgCl electrodes were inserted into the reservoir on each side of the membrane. A water column was attached to the top port of the diffusion cell, which was otherwise sealed, and used to apply a known pressure gradient across the membrane. The streaming currents generated under various pressure gradients were measured using a Keithley picoammeter 6485 with a salt solution of equal ionic concentration (50 mM, pH 3) in each reservoir.

NMR self-diffusion measurements: Approximately 5 cm x 5 cm of membrane material was soaked in 100 mM LiCl for 24 hours. The membrane was then removed from solution and dabbed dry using cleanroom grade wipes to remove bulk liquid before being rolled up and inserted into a 5 mm diameter glass NMR tube. Measurements of self-diffusion were performed on a Bruker Avance III spectrometer operating at a proton Larmour frequency of 500.13 MHz using a Bruker Prodigy Cryoprobe. Proton 90° times of 10.8 μs and 15.0 μs were used for 100 mM LiCl solutions without and with the CNT membrane, respectively, and a recycle delay of 10 seconds was used for both samples. ^{7}Li experiments were conducted at an operating frequency of 194.35 MHz using 90° times of 13.55 μs and 14.0 μs for 100 mM LiCl solutions without and with the CNT membrane, respectively, and a recycle delay of 60 seconds for both samples. Pulsed field gradient (PFG) stimulated-echo experiments were performed using the standard bipolar gradient LED pulse sequence (ledbpgp2s) at 298.15 K. All samples were thermally equilibrated for 30-60 minutes prior to analysis. Gradient pulse durations (δ) of 700 and 1600 μs and diffusion delay times (Δ) of 50 and 100 ms were used for the ^{1}H and ^{7}Li PFG experiments, respectively.

Self-diffusion coefficients were extracted from the PFG data by regression fitting of the integrated NMR signal intensity versus applied gradient strength (Figure S10). A total of 32 experiments were performed for all PFG measurements.[4]

Molecular dynamics simulations and self-diffusion calculations: All molecular dynamics (MD) simulations were performed using the program CHARMM (version 41b1).[5] We utilized the polarizable Drude force field[6] with the inclusion of the cation-π interaction that has been shown to provide a consistent description of the ion solvation in CNTs as observed with first-principles molecular dynamics simulations.[7] The confined solutions were modelled using a supercell with a dimension of a = b = 21.17 Å, c = 66.83 Å, consisting of an ion pair (LiCl and KCl)[8] and 214 SWM4 waters[9] solvated in a (19,0) CNT with a diameter of 1.5 nm. In addition, simulations of the bulk solutions were carried out for comparison, where an ion pair was solvated in a cubic box consisting of 558 SWM4 water molecules at the experimental density of 1 g/mL. The energy of the systems was minimized by using a combination of steepest descents (100 steps) and adopted basis Newton-Raphson (1000 steps) methods. The MD simulations of the confined solutions were performed in the NVT ensemble using a Velocity Verlet integrator and a Nose-Hoover thermostat at 298.15 K. A characteristic response time for the thermostats of $\tau = 0.1$ ps was used for all atoms and $\tau = 0.005$ ps for Drude particles. Here, electrostatics was treated using Particle Mesh Ewald summation with an 18 Å real space cutoff and a 1 Å grid,[9] and SHAKE algorithm was used to constrain bonds containing hydrogens.[10] Bulk simulations were performed using an NPT ensemble with the same parameters as above but also coupled to a pressure bath at 1 atm. A timestep of 1.0 fs was used for all simulations. Analysis of transport and solvation of ions and water confined in CNTs was carried out using statistics collected over four independent 10 ns

simulations. Self-diffusion coefficients of ions and water were computed from the mean-squared displacement (MSD) of the species.

Potential of mean force simulations: The potential of mean force (PMF) for the ion transport through a 1.5 nm CNT was determined by using well-tempered metadynamics (WT-MetaD) simulations. Here, WT-MetaD simulations enable sampling of rare events by depositing a history-dependent bias energy in the form of the Gaussian potential to the system's free energy landscape through a predetermined collective variables (CVs).[11] We note that the use of WT-MetaD simulations has been shown to lead to asymptotic convergence of the PMF.[12] Two collective variables were selected to facilitate the convergence of the PMF calculations.[13] These include the center-of-mass (COM) distance between the ion and CNT along the CNT direction and the COM distance along the radial direction (see Figure S1). Three replicas of WT-MetaD simulations were carried out spanning 250 ns for each ion (Ca^{+2}, Na^{+}, K^{+} and Cl^{-}). The 1D PMF was then obtained from the minimum free energy path derived from the averaged 2D PMFs. The Gaussian bias energy was deposited every 500 steps with a height of 0.12 kcal/mol and widths of 0.6 nm and 0.2 nm for the CV that describes axial and radial distance, respectively. The height of the Gaussian potential was tempered with a bias factor of 10. The simulations were performed using the LAMMPS package[14] patched with a version of PLUMED 2.5.2.[15] The OPLS-AA force fields[16] were employed in all simulations.

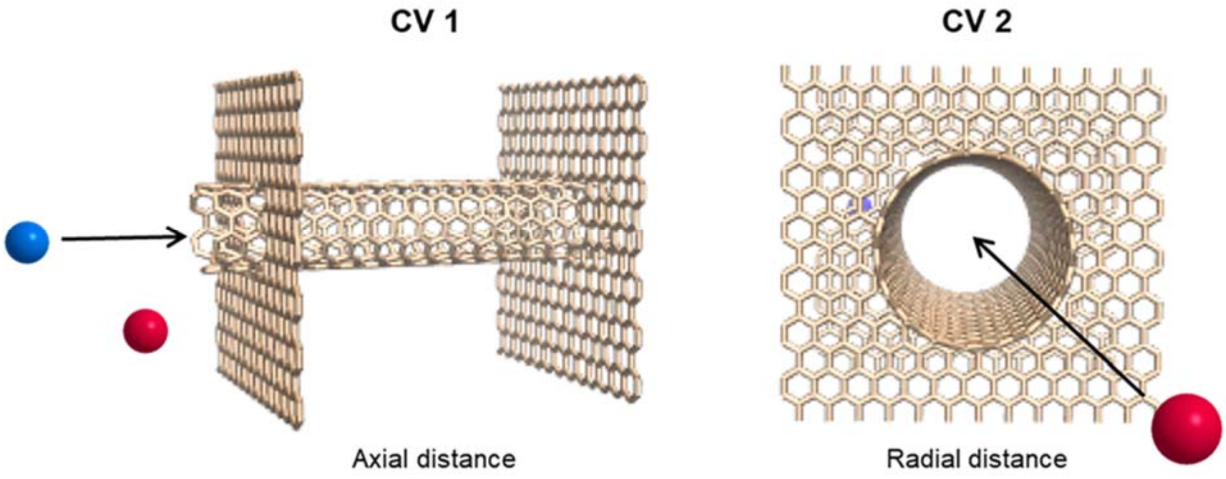

Figure S1. Definitions of the collective variables (CV 1 and CV 2) used for the description of the ion transport through a CNT.

Statistical analysis: Unless otherwise described, data is reported as the mean ± the standard deviation from at least three independent repeated runs. Exact sample size for each case is reported in the corresponding figure caption or methods section. Detection limit of the conductivity probe is defined as three times larger than the standard deviation found from at least three repeated runs under conditions where no membrane transport is expected (ex. solid piece of Kapton in place of the membrane). Where specified, best fit parameters are obtained from fitting the experimental data to the expected trend ± the error in the fit quality using the built-in fitting function in the software OriginPro.

Supplementary Text

1. Currently accepted controls in the CNT membrane literature

In this work, we developed novel controls to rule out the presence of membrane defects with diameters equal to or smaller than the CNT nanochannels, which is essential to fundamental transport studies through CNT membranes. Many groups have reported evidence of defect free transport through CNT membranes, yet these previous tests fell short of demonstrating the absence of leaky transport pathways with sizes smaller than or equal to the CNT pores. Direct imaging approaches typically do not supply either the required resolution or sample a large enough area to address nanoscale defects. Strategies based on the filtration of particles from solution, or gas diffusion scaling, rely primarily on the size or charge of the transporting pores, and therefore cannot distinguish between CNTs and defects with similar properties. Some control experiments look at transport properties prior to uncapping CNTs, but subsequent steps to open the CNTs often introduce a risk of new defect formation, as etching is rarely selective for CNTs and not all matrices provide solid filling across the entire membrane thickness. The observation of changes in selectivity following CNT tip functionalization, another common control experiment, cannot rule out the possibility that transport through nearby nanoscale defects is also affected by the same chemical modification. Matching membrane pore size distribution with the known CNT diameter distribution provides one promising strategy, however, this has been rarely performed to date. Table S1 provides a non-exhaustive summary of controls which have been reported and generally accepted thus far in the field.

Table S1. Non-exhaustive summary of control experiments used in the literature as evidence of transport through CNT pores. In nearly all cases, defects with dimensions comparable or smaller than the employed CNTs cannot be conclusively ruled out as transport pathways.

References	No transport before opening CNTs	Imaging of membrane cross-section	Pressure independent gas permeance and/or Knudsen selectivity	PEG, protein, nano-particle filtration	Exclusion of charged ions or current rectification	Selectivity change with CNT tip functional-ization	No transport with internally blocked CNTs
Zhang[17]	yes	yes	yes	-	-	-	-
Hinds[18]^	yes	yes	yes	-	yes	yes	-
Du[19]&	-	yes	-	yes	yes	-	-
Baek[20]	yes	yes	-	-	-	-	-
Park[21]	-	-	-	yes	yes	-	-
Krishnakumar[22]	yes	-	-	yes	yes	yes	-
Mi[23]	-	yes	yes	-	-	-	-
Zhang[24]	-	yes	yes	-	-	-	-
Majumder[25]	yes	yes	-	yes	-	yes	-
Majumder[26]*	yes	yes	yes	-	-	-	-
Wu[27]	yes	yes	-	-	yes	-	-
Kim[28]	yes	yes	yes	yes	yes	-	-
Fornasiero[29]	yes	yes	yes	yes	yes	-	-
Holt[30]	yes	yes	yes	yes	yes	-	
This work	yes	yes	yes	yes	yes	-	yes

*Functionalization of the inner CNT wall resulted in a loss of ultrafast water flow, ^Pore size distribution from porosimetry matched well the known CNT diameter distribution, &Dye molecules were mixed into the epoxy matrix during the membrane preparation to confirm absence of flow through cracks in the epoxy matrix, which otherwise would release trapped dye molecules.

2. Summary of literature data shown in Fig 2C of the main manuscript

Table S2. List of the references used to generate Figure 2C. References marked with an ^ indicate that the diffusion constant was calculated from mobility by applying the Einstein relation. Hydrated ion diameters from Marcus[31] were used for standard electrolytes, and an * indicates diameter estimates based on chemical structure.

Label	Citation	Ion/Salt	Ion/Salt Diameter (nm)	Pore type
		Experimental, Transport Diffusion		
1	Ref[27]	KCl	0.66	CNT
2	Ref[26]	KCl	0.66	CNT
3	Ref[32]^	Cl	0.68	Graphene Slit
4	Ref[32]^	K	0.66	Graphene Slit
5	Ref[32]^	Na	0.72	Graphene Slit
6	Ref[32]^	Li	0.76	Graphene Slit
		Experimental Self-Diffusion		
7	Ref[33]	Bmim	0.80*	Mesoporous Carbon
8	Ref[34]	Omim	1.20*	CNT
9	Ref[34]	Bmim	0.80*	CNT
		Simulation, Self-Diffusion		
10	Ref[35]	Dmim	1.63*	Graphene Slit
11	Ref[35]	Cl	0.68	Graphene Slit
12	Ref[36]	C_4mim	0.80*	CNT
13	Ref[36]	C_4mim	0.80*	CNT
14	Ref[36]	C_4mim	0.80*	CNT
15	Ref[37]	C_2C_1IMCl	0.50*	CNT
16	Ref[37]	C_2C_1IMCl	0.50*	CNT
17	Ref[37]	C_2C_1IMCl	0.50*	CNT
18	Ref[37]	C_2C_1IMCl	0.50*	CNT
19	Ref[37]	C_2C_1IMCl	0.50*	CNT
20	Ref[38]	K	0.66	Smooth Cylindrical Pore
21	Ref[38]	Cl	0.68	Smooth Cylindrical Pore
22	Ref[39]	Na	0.72	Graphene Slit
23	Ref[39]	Cl	0.68	Graphene Slit
24	Ref[39]	Na	0.72	Graphene Slit
25	Ref[39]	Cl	0.68	Graphene Slit

3. Vertically aligned CNT forest properties

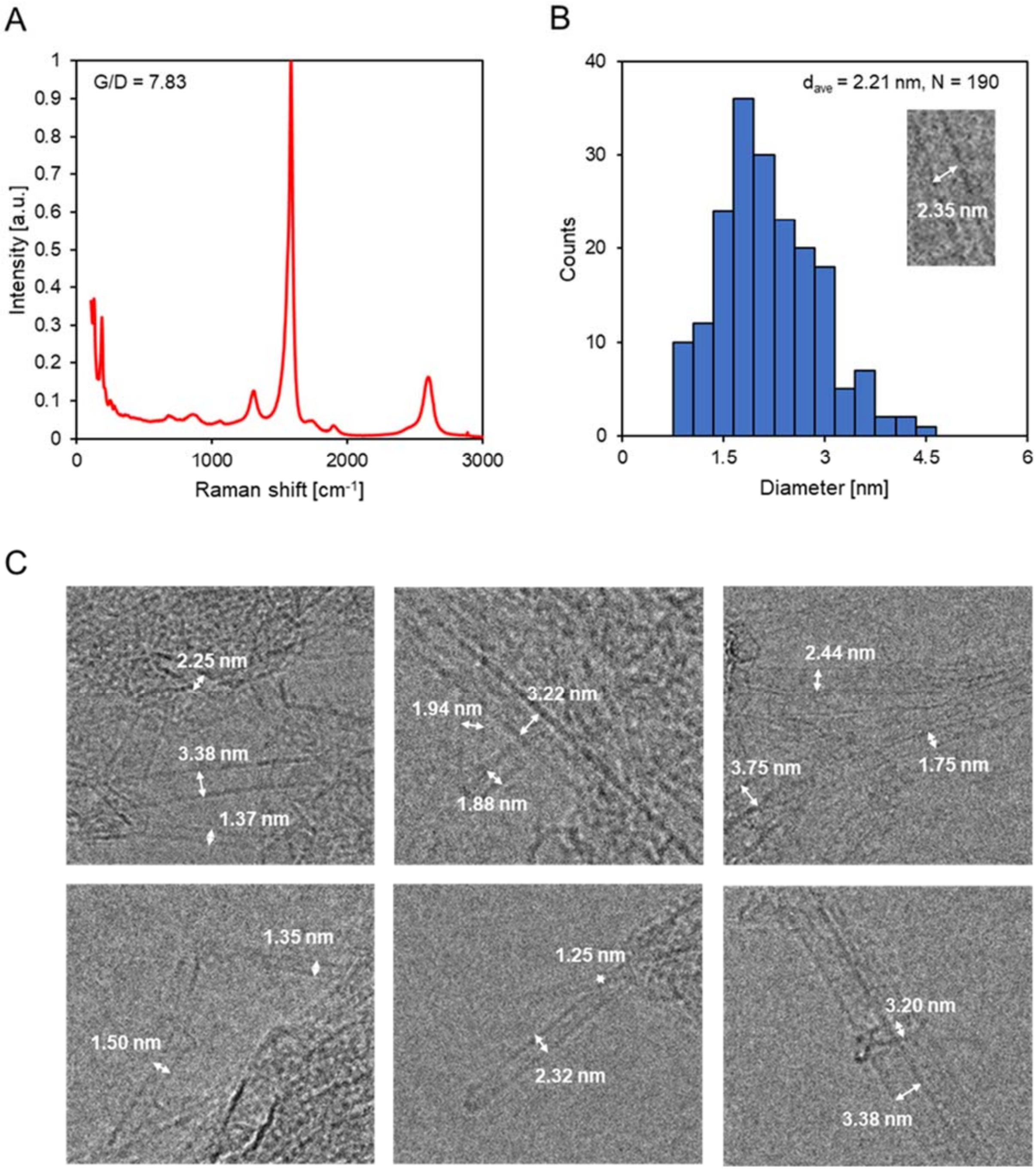

Figure S2. A) Representative Raman spectra and B) diameter distribution generated from TEM (inset) taken from CNT forests used in this work. C) Selected TEM images from the dataset used to produce the histogram in panel B.

4. Parylene matrix type does not influence transport behavior

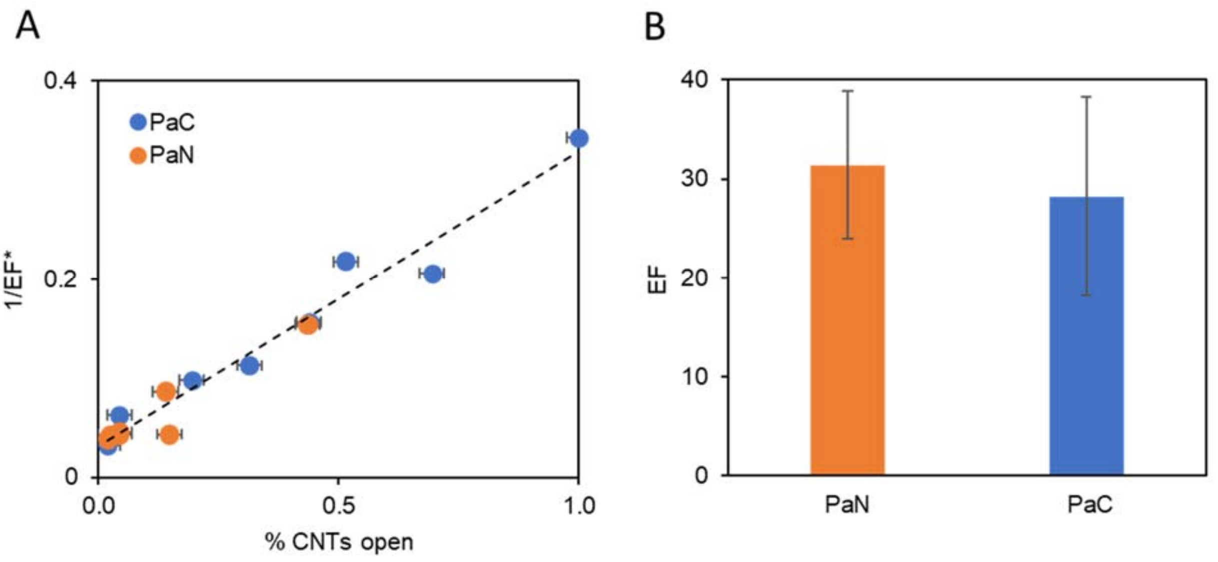

Figure S3. A) *1/EF** is plotted vs percentage of open CNTs, which was calculated from the same N_2 opening curve used in the main text. Membranes made from both parylene-N and -C matrix materials fall along the same line, indicating comparable transport performance. B) Extrapolated *EF* for KCl using only the parylene-N dataset (orange) and parylene-C dataset (blue). Error bars represent standard error from the linear fit of *1/EF** vs % CNTs open.

5. Diffusion cell

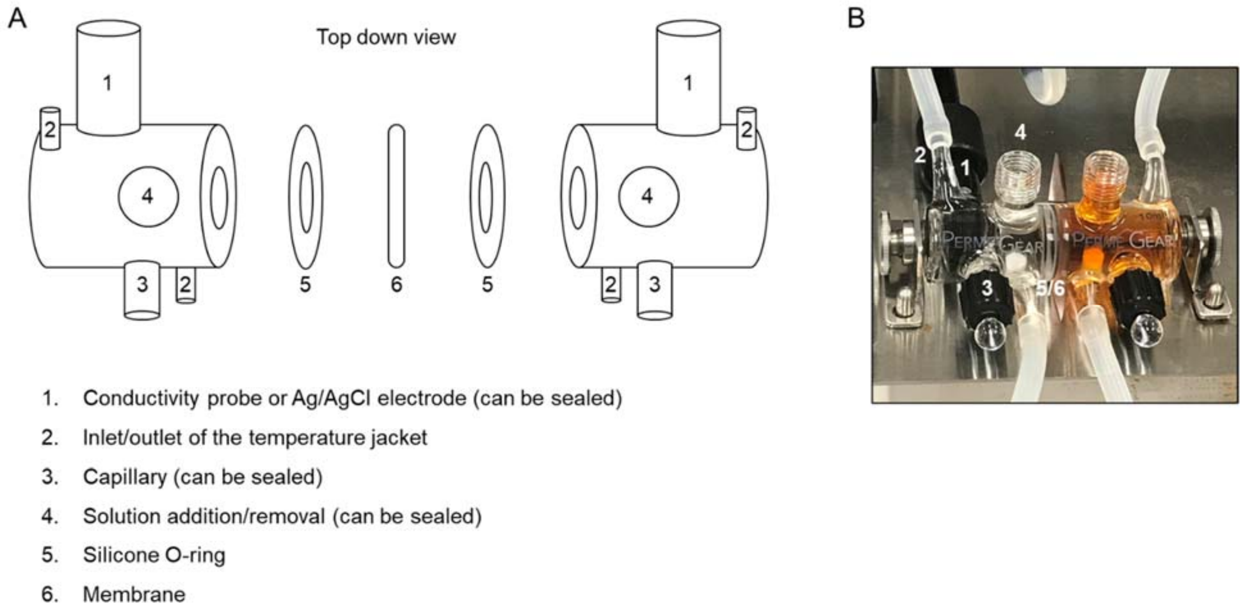

Figure S4. A) Schematic of the custom designed diffusion cell used for transport experiments. B) Image of diffusion cell shown with capillary ports in the sealed configuration.

6. Control membrane C1 characterization

As demonstrated in the main manuscript, no transport is observed through C1 membranes in which CNTs have been uncapped prior to parylene-N infiltration, indicating that the CNT interiors are clogged with polymer. SEM characterization of CNT forests after uncapping with low power plasma etching shows minimal changes to the forest morphology. Measured G/D values by Raman spectroscopy on uncapped CNT/parylene composites prior to the final opening step are comparable to composites made from pristine CNT forests, with a decrease only in the top few microns (which include also the ~ 1 micron-thick excess parylene on the membrane surface). This region is eventually thinned by the final etching process in both standard and control membranes. Thus, these modest changes in nanotube graphitization level due to the plasma treatment in C1 controls are not expected to have a significant impact on measured transport rates.

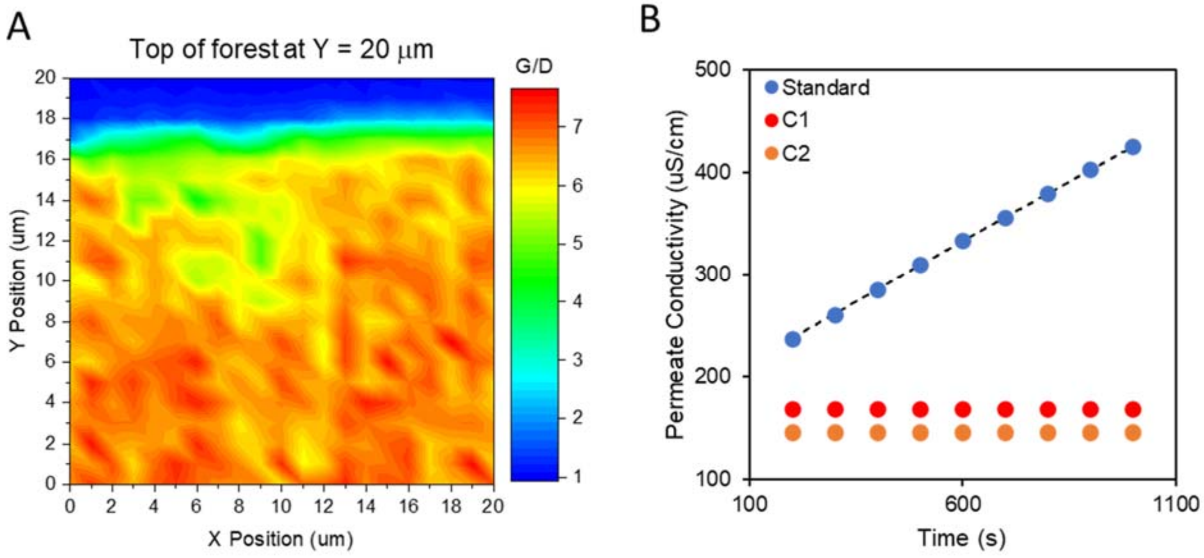

Figure S5. A) Area map showing the ratio of G/D peaks in the Raman spectra for the cross section of a CNT/parylene composite made from an uncapped CNT forest prior to the final etch step. Sample is oriented with the CNT tips located at the top (Y = 20 ± 1 μm). B) Permeate conductivity vs time under a 50 mM KCl concentration gradient at pH 3, for a standard membrane (blue), control type 1 (red) and control type 2 (orange). Black line is the fit to Equation S3.

7. Quantification of boundary layer resistance

Diffusion measurements were performed by bathing the two surfaces of a CNT membrane with salt solutions of different concentrations. The experimentally obtained ion fluxes include contributions from both the membrane and the boundary layer resistances in the solution near the membrane surfaces. Despite stirring of the two salt solutions, a “stagnant” film exists (the boundary layer) next to the membrane surfaces in which the salt concentration change and solution resistance to salt transport are concentrated. The salt transport through the boundary layer is modeled as diffusion through a stagnant layer with a thickness δ that depends on the stirring rate of the system (held constant for all experiments in this work). These boundary layer resistances on the membrane surfaces are in series with the intrinsic membrane resistance. Conservation of species implies that the salt flux across each boundary layer must be equal to the flux through the membrane, F_{meas}. Therefore,

$$F_{meas} = \frac{D_{bulk}}{\delta}\left(C_{bulk}^{High} - C_{sol}^{I}\right) = \varphi\frac{D_{mem}}{\tau L_{mem}}(C_{mem}^{I} - C_{mem}^{II})$$
$$= \frac{D_{bulk}}{\delta}(C_{sol}^{II} - C_{bulk}^{Low}) \qquad \text{(S5)}$$

where D_{bulk} is the salt diffusion coefficient in the bulk, C_{bulk}^{High} and C_{bulk}^{Low} are the concentrations of the high and low salinity solutions, respectively, in the two chambers of the diffusion cell, C_{sol}^{I} and C_{sol}^{II} are the salt concentration of the solution next to the membrane surfaces, C_{mem}^{I} and C_{mem}^{II} are the salt concentrations in the membrane next to the surface, L_{mem} is the membrane thickness, δ is the boundary layer thickness (assumed to be equal at the two sides of the membrane), τ and φ are the tortuosity and porosity of the membrane, respectively. The membrane (R_{mem}) and boundary layer (R_{BL}) resistances correspond to the inverse of the terms multiplying the concertation differences in (S5):

$$R_{mem} = \frac{\tau L_{mem}}{\varphi D_{mem}} \tag{S6}$$

$$R_{BL} = \frac{\delta}{D_{bulk}} \tag{S7}$$

Thermodynamic equilibrium is typically assumed between solution and membrane phases at the membrane surface, and therefore C_{bulk}^{I} and C_{bulk}^{II} can be related to C_{mem}^{I} and C_{mem}^{II} with $K_{H2O-mem}$, the partition coefficient between bulk solution and the membrane:

$$C_{mem}^{i} = K_{H2O-mem} C_{bulk}^{i} \qquad i = I, II \tag{S8}$$

Equation (S5) can therefore be rewritten as

$$\frac{F_{meas}}{\frac{D_{bulk}}{\delta}} = F_{meas} R_{BL} = \left(C_{bulk}^{High} - C_{sol}^{I}\right) \tag{S9}$$

$$\frac{F_{meas}}{\varphi \frac{K_{H2O-mem} D_{mem}}{\tau L_{mem}}} = F_{meas} \frac{R_{mem}}{K_{H2O-mem}} = (C_{sol}^{I} - C_{sol}^{II}) \tag{S10}$$

$$\frac{F_{meas}}{\frac{D_{bulk}}{\delta}} = F_{meas} R_{BL} = (C_{sol}^{II} - C_{bulk}^{Low}) \tag{S11}$$

Summation of these three terms allows to eliminate the interfacial concentrations and express the measured flux in terms of the bulk solution concentrations and the membrane and boundary layer resistances:

$$F_{meas} = \frac{1}{\frac{R_{mem}}{K_{H2O-mem}}+2R_{BL}}\left(C_{bulk}^{High} - C_{bulk}^{Low}\right) = \frac{1}{\frac{\tau L_{mem}}{\varphi D_{mem} K_{H2O-mem}}+2\frac{\delta}{D_{bulk}}}\left(C_{bulk}^{High} - C_{bulk}^{Low}\right) \qquad \text{(S12)}$$

In the manuscript, we defined *EF** as the ratio of the measured flux, $F_{meas.}$, to that expected assuming bulk diffusion in the membrane pores, F_{bulk}.

$$F_{bulk} = \varphi \frac{D_{bulk}}{\tau L_{mem}}\left(C_{bulk}^{High} - C_{bulk}^{Low}\right) \qquad \text{(S13)}$$

Therefore, 1/*EF** is equal to:

$$\frac{1}{EF^{*}} = \frac{D_{bulk}}{D_{mem} K_{H2O-mem}} + 2\varphi \frac{\delta}{\tau L_{mem}} = \frac{1}{EF} + 2\varphi \frac{\delta}{\tau L_{mem}} \qquad \text{(S14)}$$

which is Equation 1 of the main text. Using this linear relation between 1/*EF** and the membrane porosity φ, 1/*EF* can be obtained from the intercept and δ from the slope and the known structural properties of the membrane (thickness and tortuosity). R_{BL} is then calculated from δ with Equation (S7)**Error! Reference source not found.**. These equations accounting for the boundary layer do not make any assumption on the type of membrane (which is essentially a black box) and are generally valid, even when flow inside the membranes occurs through nanopores.

Table S3. Polycarbonate membrane characteristics. Average diameter and pore density were measured via SEM imaging. Reported diameters include a 9.4 nm correction factor to account for the deposition of a gold layer which was required to collect high resolution images. Thickness was evaluated using a pin caliper. Membrane tortuosity is assumed to be 1.

Membrane Number	Average Pore Diameter (nm)	Membrane Thickness (µm)	Pore Density (cm^{-2})
PC-1	106	6.7	3.7×10^8
PC-2	415	27	1.3×10^8
PC-3	57	5.5	8.4×10^8
PC-4	107	5.5	4.4×10^8
PC-5	91	5.5	8.1×10^8
PC-6	34	6.0	7.6×10^8

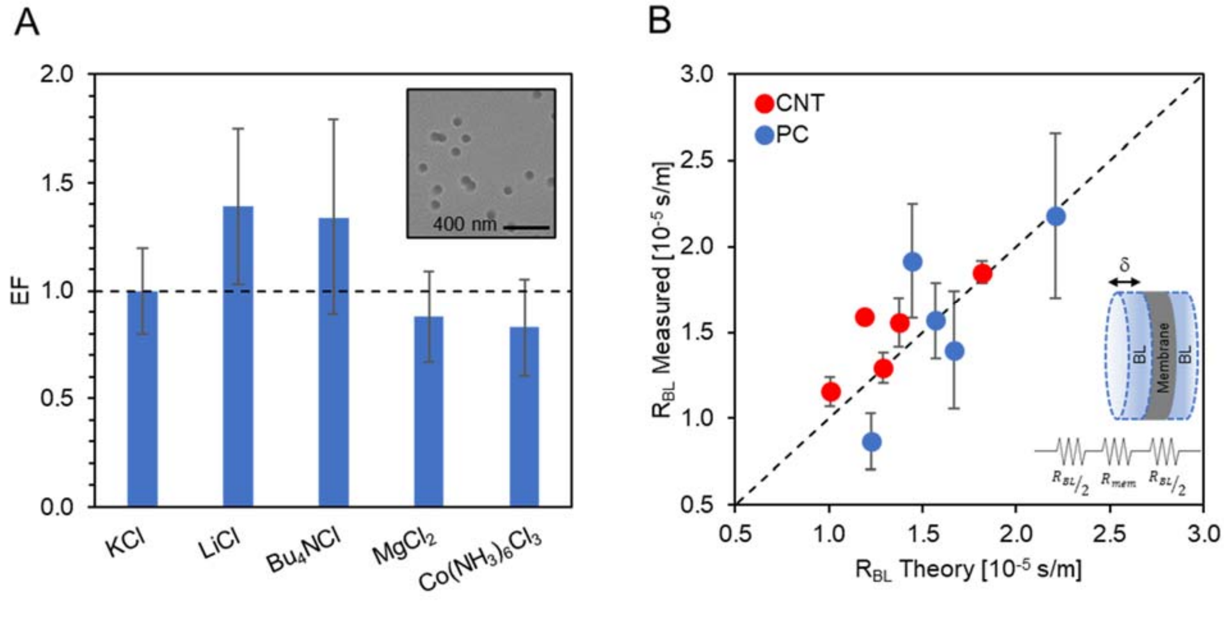

Figure S6. A) Diffusion enhancement factor extrapolated from a linear fit of *1/EF** vs the reciprocal of membrane resistance for a set of 5 polycarbonate track etched membranes with varying membrane resistance. *EF* is equal to unity indicating bulk like diffusion through the membranes. Inset: representative SEM image from which the pore diameter and density was calculated. B) Boundary layer resistance measured experimentally from the same polycarbonate dataset (blue) and from a set of CNT membranes with varied membrane resistance (red). The horizonal axis represents the theoretical prediction that the ratio of R_{BL} for two salts scales with the ratio of their diffusion coefficients to the 2/3 power.[40] Predicted R_{BL} is scaled off of LiCl. Inset: diagram showing the boundary layer resistance geometry and equivalent resistance diagram. Error bars are taken from the quality of the linear fit to the plot of *1/EF** vs $1/R_{mem}$.

8. Summary of measured salt permeability in our CNT membranes vs bulk

Table S4. Bulk diffusion constant for tested salts along with permeability ($D_{mem}K_{H2O-mem}$) and EF in our CNT membranes.

Salt	D_{bulk} $[m^2/s]$	$D_{mem}K_{H2O-mem}$ $[m^2/s]$	EF
HCl	3.34E-09	1.21E-07	36.36
LiCl	1.37E-09	3.51E-08	25.65
KCl	1.99E-09	6.08E-08	30.57
NaCl	1.61E-09	4.61E-08	28.60
CsCl	2.04E-09	6.25E-08	30.60
$CaCl_2$	1.34E-09	2.70E-08	20.15
$MgCl_2$	1.25E-09	2.37E-08	18.99
K_2SO_4	1.53E-09	3.64E-08	23.73
$MgSO_4$	8.50E-10	1.57E-08	18.46
Pr_4NCl	9.48E-10	2.17E-08	22.93
Et_4NCl	1.22E-09	3.08E-08	25.22
Bu_4NCl	8.21E-10	1.87E-08	22.76
$K_4Fe(CN)_6$	1.47E-09	2.48E-08	16.89
$K_3Fe(CN)_6$	1.51E-09	2.94E-08	19.48
$Co(NH_3)_6Cl_3$	1.55E-09	2.42E-08	15.64

9. Differences between transport and self-diffusion coefficient

It is important to note that the NMR and MD simulation analysis provides the self-diffusion constant (associated with Brownian molecular motion) and not the transport-diffusion constant (associated with a macroscopic mass transfer), and are performed in the absence of a concentration gradient. These two diffusivities are in general distinct quantities and become identical for thermodynamically ideal systems. In addition, what we have experimentally measured by wetting the two surfaces of the membranes with solution of different salinities is an "effective" salt permeability, which includes transport-diffusion constant, partition coefficient, and possibly contributions from other unaccounted transport modes. As an illustrative example, a concentration gradient could drive bulk fluid motion by diffusion osmosis, which could result in an effective, enhanced permeability. Due to thermodynamic non-ideality, the still incomplete understanding of transport phenomena under nanoscale confinement, and the possibility of contributions from other unaccounted transport modes, the "effective" transport-diffusion constant in our measurements could differ significantly from the ion self-diffusion constant in a CNT membrane.

10. Diffusio-osmosis does not explain the enhanced ion transport

As a possible explanation of the enhanced diffusive ion transport in our CNT membranes, we hypothesized a large contribution from diffusio-osmotic bulk flow. To match the observed ion transport enhancement, the magnitude of the volumetric flow rate of water, Q_{w-exp}, should be equal to

$$Q_{w-exp} = \frac{F_{meas} - F_{bulk}}{C_F} \tag{S15}$$

where F_{meas} is the measured salt flux through the membrane, F_{bulk} is the salt flux expected through our membrane assuming bulk diffusion inside the CNTs and no water flux, and C_F is the salt concentration in the feed reservoir. Values given by this analysis suggest water flux would have to be several orders of magnitude above those we see in our system. Additionally, to enhance diffusive transport, the direction of this water flux would have to be into the permeate reservoir. Water flux in our system is instead into the feed reservoir and is consistent with standard diffusion from high to low concentration. The increase in water flux with increasing ion size suggests that a small amount of selectivity may be present in our membranes for the larger ions tested, despite working at pH 3.

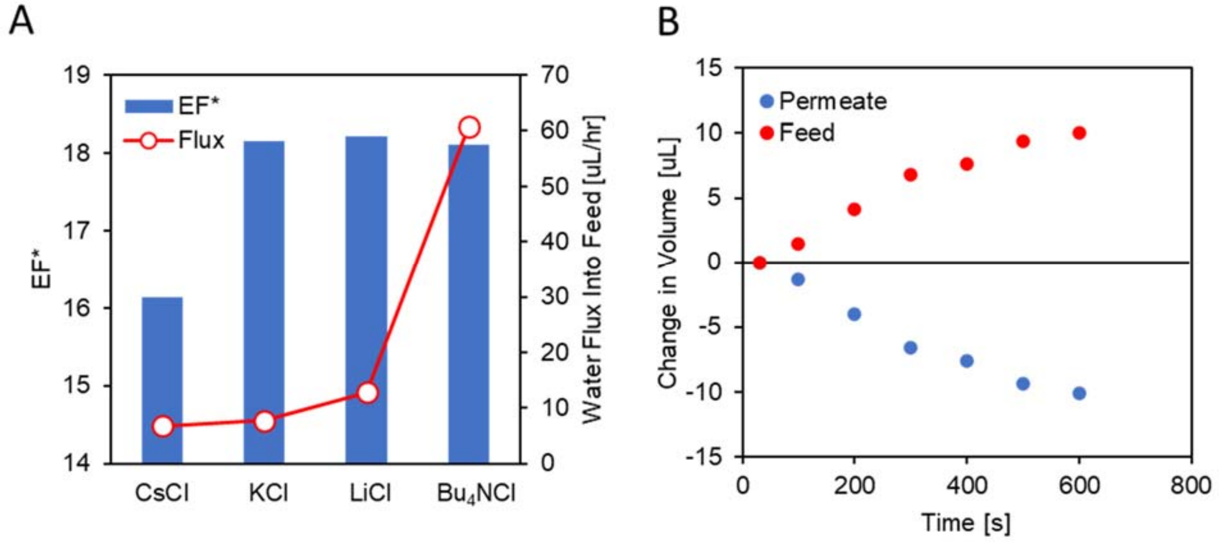

Figure S7. A) *EF** for a standard CNT membrane with approximately 5% of the CNTs open (blue, left axis) and water flux into the feed reservoir (red, right axis). B) Change in the water volume of the permeate reservoir (blue) and feed reservoir (red) vs time for the same membrane, under a gradient of 50 mM Bu_4NCl at pH 3.

11. Measured membrane Zeta potential is too low to explain enhanced transport

To further support the conclusions from the previous section, we also estimated the diffusion-osmotic coefficients for a charged nanochannel with nonzero slip. Following equations described by Mouterde et al.[41] with the simplification that only fixed charges are present at the channel wall, we calculate D_{DO}, the diffusio-osmotic mobility, as

$$D_{DO} = \frac{k_B T}{2\pi\eta\ell_B}\left(\frac{b}{\lambda_D}\left[\sqrt{1+\chi^2}-1\right] + ln\left[\frac{\sqrt{1+\chi^2}+1}{2}\right]\right) \tag{S16}$$

where k_B is the Boltzmann constant, T is the temperature, η is the viscosity of water, ℓ_B is the Bjerrum length, λ_D is the Debye length, b is the slip length. χ is defined as

$$\chi = \sinh\frac{e\psi}{2k_B T} \tag{S17}$$

where ψ is the potential at the CNT wall and e the electron charge. Assuming a weak potential, the zeta potential, ζ, is related to ψ and the slip length by the equation below.

$$\zeta = \psi\left(1+\frac{b}{\lambda_D}\right) \tag{S18}$$

In the case of unequal cation and anion mobilities (μ_+ and μ_-, respectively), the supplemental mobility term

$$D_{DO}^{supp} = -\beta\frac{k_B T\epsilon}{e\eta}\zeta \tag{S19}$$

must be added to Equation S16, with $\beta = (\mu_+ - \mu_-)/(\mu_+ + \mu_-)$. We estimated the diffusio-osmotic transport enhancement EF_{DO} with respect to bulk as

$$EF_{DO} = \frac{D_{bulk} + (D_{DO} + D_{DO}^{supp})}{D_{bulk}} \tag{S20}$$

Figure S8 shows the predicted EF_{DO} as a function of ζ and ψ as well as the relationship between the latter two properties for three different slip lengths. For a fixed ψ, large slip lengths can result in enormous amplifications of the ionic transport (Figure S8B). However, for the measured Zeta potential of our CNT membranes (a few mV), the contribution of diffusio-osmosis would be negligible even for large slip lengths (Figure S8A).

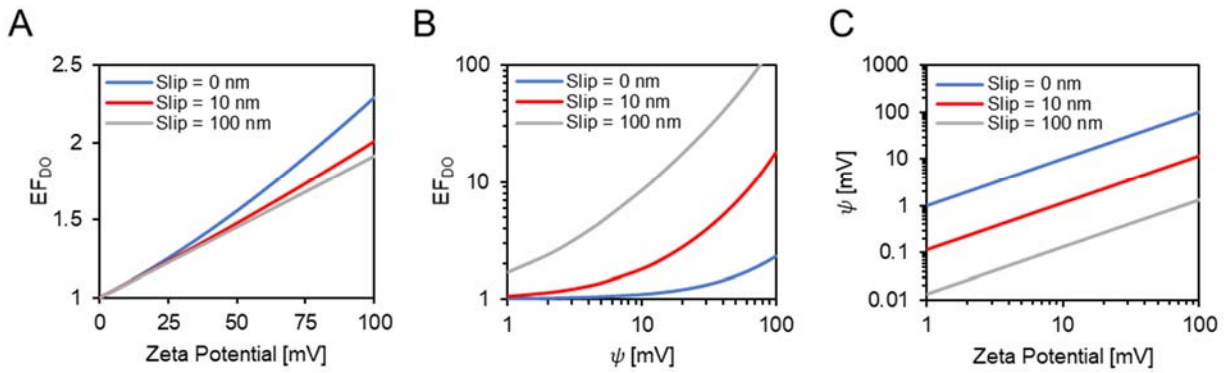

Figure S8. Calculated EF_{DO} as a function of Zeta potential (A) and surface potential (B) using Equation S20 for a slip length of 0 (blue), 10 nm (red), and 100 nm (grey). (C) Plot showing relationship between Zeta potential and surface potential using Equation S18. For all panels, $\lambda_D \sim$ 6 nm (corresponding to 50 mM) and β was set to -1 to maximize EF_{DO}.

12. Negligible effect of charges at the CNT opening

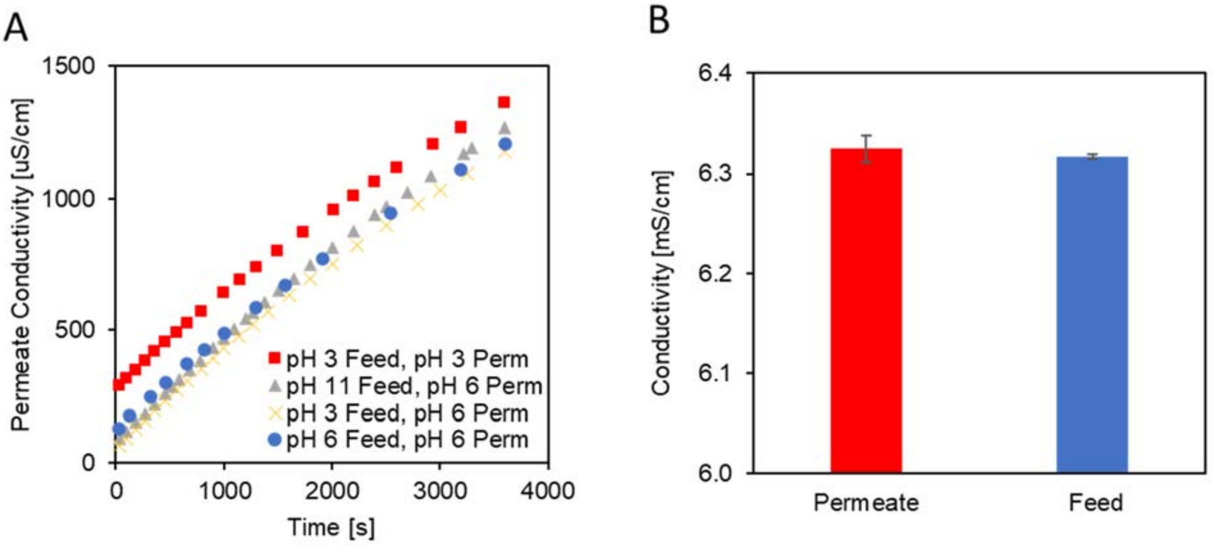

Figure S9. A) Permeate conductivity vs time for a single standard CNT membrane under a 50 mM KCl gradient for various arrangements of pH in the feed and permeate reservoirs. The slope is constant across all pH values tested indicating that surface charge from carboxyl groups at the pore entrance (which would be neutralized at low pH) does not play a significant role in the diffusive transport rates. B) Conductivity of the feed and permeate after an unbuffered 50 mM KCl solution is filtered through a standard CNT membrane under a 2-psi pressure gradient showing no ion rejection (conductivities are within 0.12%). Error bars represent standard deviation from repeated measurements with the conductivity probe. With these unbuffered solutions, the CNT surface charge is expected to play a larger role than during the ion diffusion studies with 50 mM salt solutions at pH 3, since at these conditions the carboxylic groups eventually present at the CNT tips would be fully protonated (pH ≤ pKa ~ 4.5).

13. NMR Self-Diffusion Measurements

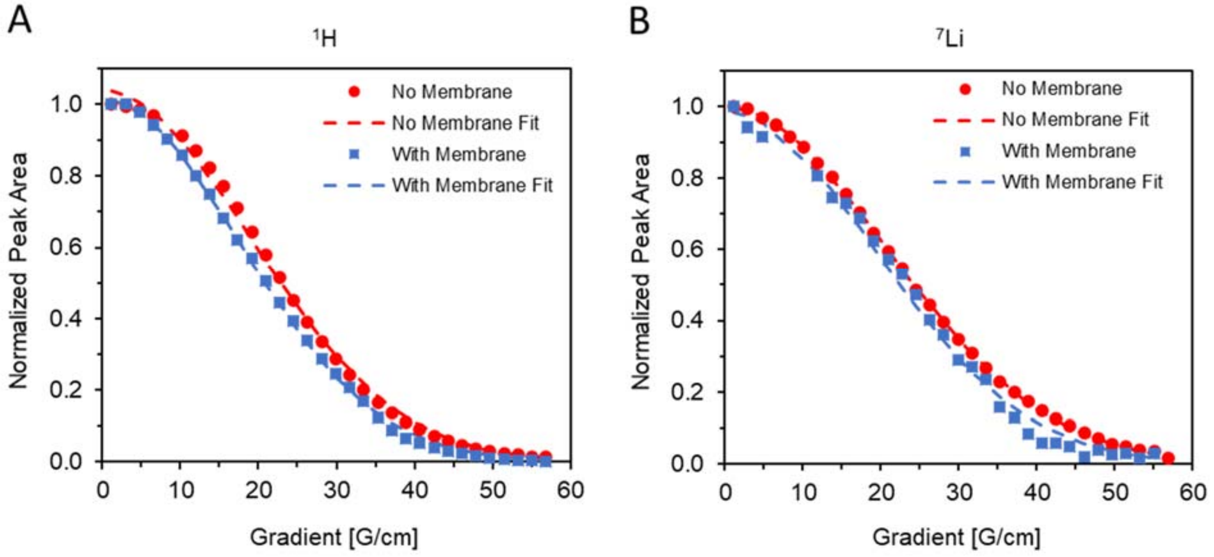

Figure S10. Normalized peak area vs magnetic field gradient strength from PFG NMR experiments for (A) ^{1}H and (B) ^{7}Li. The results of regression fitting are also shown in dashed lines.

14. Mean squared displacement and radial distribution functions from classical simulations

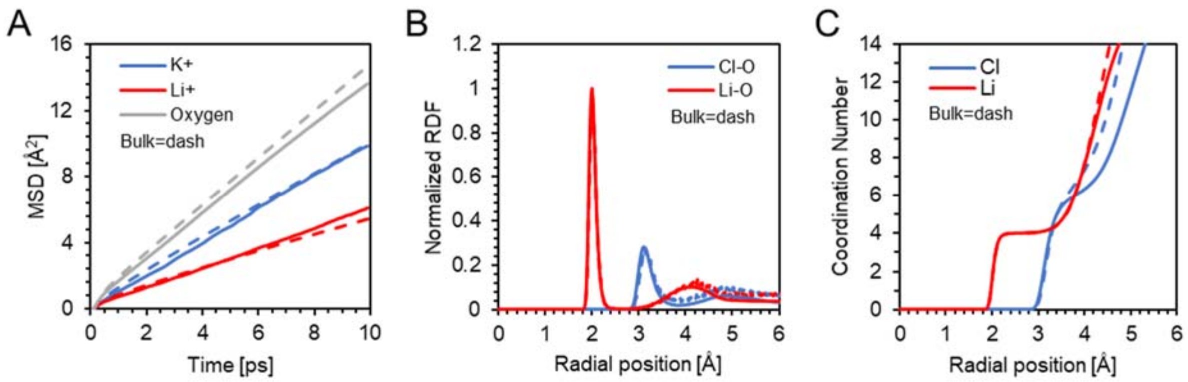

Figure S11. (A) Mean squared displacements, (B) radial distribution functions, and (C) ion coordination numbers from MD simulations of ions and water in bulk (dashed lines) and confined in a 1.5 nm diameter CNT (solid lines). For a facile comparison, ion RDFs in bulk and in confinement are normalized to the tallest Li^+ peak in the respective environment.

15. Derivation of free energy penalty to transfer salt from bulk water to the interior of a pore

Equations 2 and 3 were obtained by modifying the expression of the Gibbs free energy change to transfer an ion from water to a different solvent. In our system, the “solvent” is confined water. We refer to Chapter 4 of Marcus’ book[31] for the detailed derivation of the free energy change from water to a different solvent, and we describe here how we adapted that expression to describe confined water.

The free energy of transfer for an ion i from bulk water to a solvent (here, confined water) can be expressed as the sum of the free energy requirement for transferring a neutral analog to the ion (subscript HS, hard sphere), $\Delta G^{i}_{HS,H2O-CNT}$, and the electrostatic energy to first discharge the ion in bulk water before the transfer and then charge the neutral particle to the ion valence in the solvent, $\Delta G^{i}_{el,H2O-CNT}$:

$$\Delta G^{i}_{tot,H2O-CNT} = \Delta G^{i}_{HS,H2O-CNT} + \Delta G^{i}_{el,H2O-CNT} \qquad i = cation, anion \tag{S21}$$

$$\Delta G^{i}_{tot,H2O-CNT} = RTlnK^{i}_{HS,H2O-CNT} + \frac{N_A e^2}{4\pi\varepsilon_0} z_i^2 \left[\left(\frac{1}{\varepsilon r_{i,eff}} \right)_{CNT} - \left(\frac{1}{\varepsilon r_{i,eff}} \right)_{H2O} \right] \tag{S22}$$

where $K^{i}_{HS,H2O-CNT}$ is the distribution ratio between water and CNT for a neutral sphere with the same size of the ion i, N_A is Avogadro’s number, z is the valence, ε_0 is the permittivity of vacuum, ε_{H2O} and ε_{CNT} are the relative permittivity of bulk water and water inside a CNT, respectively, and r_{eff} is the effective ion radius. Here we have assumed that the effective radius of the ion in the aqueous solution inside the CNTs is equal to that in bulk water, $r_{i,eff}$, i.e. identical ion hydration inside the CNT and in the bulk. This assumption is justified by the results

of our MD simulations for 1.5 nm wide CNTs in Figure 3B. Values for $r_{i,eff}$ in water are tabulated in the same book.

For purely steric interactions between a hard sphere of radius r_i and a cylindrical pore of radius r_{CNT}, the equilibrium partition coefficient is given by:[42]

$$K^{i}_{HS,H2O-CNT} = \left(1 - \frac{r_i}{r_{CNT}}\right)^2 \quad \text{(S23)}$$

This expression derives from the condition that, to enter a pore, a molecule must pass through the opening without striking the edge. Therefore, the center of the molecule must pass through a circle of radius (r_{CNT} - r_i) within the mouth of the pore, which is only a fraction of the total cross-sectional area of the pore πr^2_{CNT}. This fraction equals in magnitude to $K^{i}_{HS,H2O-CNT}$. As customary in the transport literature (see, for ex., Deen's hindered transport theory), r_i is set here equal to the ion Stokes radius.

To quantify the energy penalty for a salt $An^{z_{an}}_{v_{an}}Cat^{z_{cat}}_{v_{cat}}$, we combine the anion and cation free energies by accounting for the fact that one mole of salt in solution dissociates into v_{an} anion and v_{cat} cation moles:

$$\Delta G^{salt}_{tot,H2O-CNT} = v_{an}\Delta G^{an}_{tot,H2O-CNT} + v_{cat}\Delta G^{cat}_{tot,H2O-CNT} \quad \text{(S24)}$$

Inserting (S22) and (S23) into (S24) gives the electrostatic and neutral hard sphere component of the energy penalty for the salt

$$\Delta G_{el,H2O-CNT} = \frac{N_A e^2}{4\pi\varepsilon_0}\left(\frac{v_{cat} z^2_{cat}}{r_{cat,eff}} + \frac{v_{an} z^2_{an}}{r_{an,eff}}\right)(\varepsilon^{-1}_{CNT} - \varepsilon^{-1}_{H2O}) \quad \text{(S25)}$$

$$\Delta G_{HS} = -RTln\left[\left(1 - \frac{r_{cat}}{r_{CNT}}\right)^{2\nu_{cat}}\left(1 - \frac{r_{an}}{r_{CNT}}\right)^{2\nu_{an}}\right] \tag{S26}$$

which correspond to Equations 2 and 3 of the main manuscript.

Therefore, these equations account for steric effects due to ion confinement as well as the electrostatic energy contribution due to variation of the dielectric constant in confined water, but neglect changes in ion hydration and ion-CNT wall interactions that may occur inside a pore. Thus, we expect predictions from Equations 2 and 3 to become increasingly more accurate for larger diameter CNT pores, in which perturbations of the ion solvation become negligible and the solvent properties and structure are more similar to bulk water.

Peter and Hummer have computed the free-energy difference between the bulk water phase and the interior of a ~1 nm CNT for Na^+ with both continuum electrostatics and MD simulations.[43] They concluded that continuum electrostatics correctly predicts that a CNT pore of this (or larger) diameter poses a low free-energy barrier for ion insertion. This energy barrier obtained from potential of mean force (PMF) calculations amounts to ~ 3 kJ/mole. Equations (S22) and (S23) give an energy penalty of 2.54 kJ/mole for Na^+, which agrees well with the PMF calculations. The same authors point out that a major contribution to the PMF energy barrier is the restriction of the accessible space in the radial direction when the ion is inside the CNT. This contribution corresponds to Equation (S23). Note that the pore size in these calculations (i.e, 1-nm) is significantly smaller than the 2.2-nm average diameter of our CNT membranes. We expect, therefore, Equations 2 and 3 to perform even better in estimating the energy barrier for ion insertion in our larger CNTs.

16. Comparison of Equations 2 and 3 with potential of mean force simulations

To have a measure of the approximation level of Equations 2 and 3 of the manuscript, we performed potential of mean force (PMF) calculations for a series of ions to enter a 1.5 nm-wide CNT (1.16 nm inner diameter) and compared these MD results with estimates from Equations 2 and 3 applied to a single ion. These calculations are reported in Figure S12 and show that Equations 2 and 3 underestimate somewhat the energy barrier to access a CNT pore but capture correctly both the order of magnitude and the energy penalty trend with ion type.

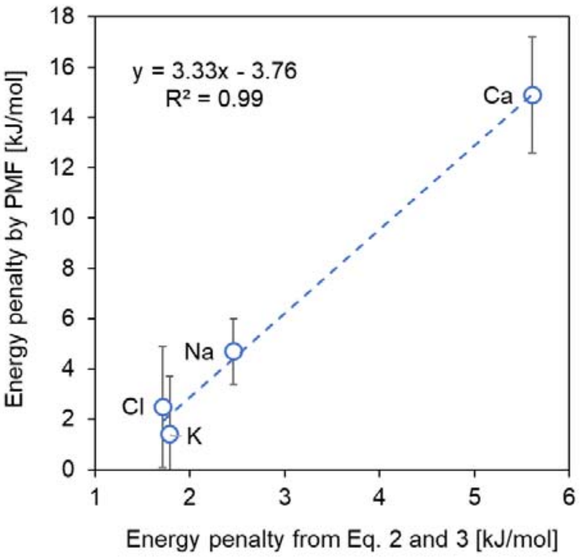

Figure S12. Comparison of the energy penalty for an ion to enter a 1.5 nm-wide CNT (1.16 nm inner diameter) from bulk solution calculated using Equations 2 and 3 of the main text and potential mean force simulations. Error bars are taken as standard deviation from triplicate simulations.

17. Comparison of membrane permeability with other membrane types

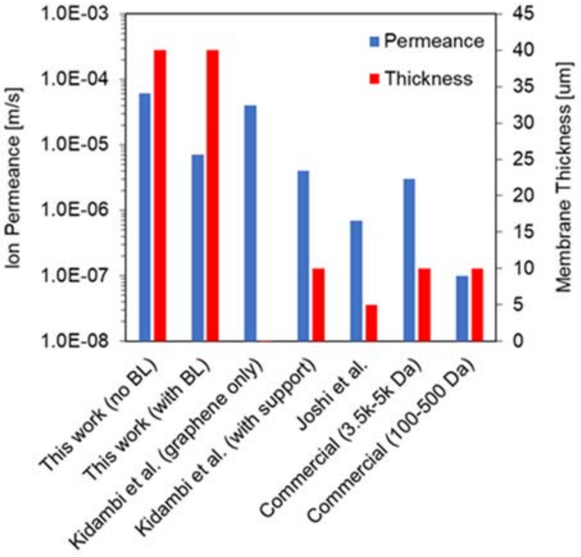

Figure S13. Comparison of permeance for either KCl or K^+ in different membrane types. The permeance for this work in the absence of a boundary layer resistance was calculated from the 1.9% open membrane by scaling to 100% CNTs open. Permeance including the boundary layer resistance was calculated from the measured flux through a 100% open membrane. Joshi et al. [44] data is for K^+ diffusion through graphene oxide membranes, Kidambi et al. [45] data is for KCl diffusion through nanoporous monolayer graphene membranes with and without inclusion of the porous support, and the permeance for the commercially available membrane is for KCl [45].

References

[1] E. R. Meshot, S. J. Park, S. F. Buchsbaum, M. L. Jue, T. R. Kuykendall, E. Schaible, L. B. Bayu Aji, S. O. Kucheyev, K. J. J. Wu, F. Fornasiero, Carbon 2020, 159, 236.

[2] S. Esconjauregui, R. S. Xie, M. Fouquet, R. Cartwright, D. Hardeman, J. W. Yang, J. Robertson, J. Appl. Phys. 2013, 113.

[3] N. Bui, E. R. Meshot, S. Kim, J. Peña, P. W. Gibson, K. J. Wu, F. Fornasiero, Advanced Materials 2016, 28, 5871.

[4] T. D. W. Claridge, in *High-Resolution NMR Techniques in Organic Chemistry*, Elsevier, Cambridge, MA 2016, 381.

[5] B. R. Brooks, C. L. Brooks III, A. D. Mackerell Jr., L. Nilsson, R. J. Petrella, B. Roux, Y. Won, G. Archontis, C. Bartels, S. Boresch, A. Caflisch, L. Caves, Q. Cui, A. R. Dinner, M. Feig, S. Fischer, J. Gao, M. Hodoscek, W. Im, K. Kuczera, T. Lazaridis, J. Ma, V. Ovchinnikov, E. Paci, R. W. Pastor, C. B. Post, J. Z. Pu, M. Schaefer, B. Tidor, R. M. Venable, H. L. Woodcock, X. Wu, W. Yang, D. M. York, M. Karplus, Journal of Computational Chemistry 2009, 30, 1545.

[6] G. Lamoureux, B. t. Roux, The Journal of Chemical Physics 2003, 119, 3025.

[7] T. A. Pham, S. M. G. Mortuza, B. C. Wood, E. Y. Lau, T. Ogitsu, S. F. Buchsbaum, Z. S. Siwy, F. Fornasiero, E. Schwegler, The Journal of Physical Chemistry C 2016, 120, 7332.

[8] H. Yu, T. W. Whitfield, E. Harder, G. Lamoureux, I. Vorobyov, V. M. Anisimov, A. D. MacKerell, B. Roux, Journal of Chemical Theory and Computation 2010, 6, 774.

[9] T. Darden, D. York, L. Pedersen, The Journal of Chemical Physics 1993, 98, 10089.

[10] J.-P. Ryckaert, G. Ciccotti, H. J. C. Berendsen, Journal of Computational Physics 1977, 23, 327.

[11] A. Barducci, G. Bussi, M. Parrinello, Phys Rev Lett 2008, 100, 020603.

[12] J. F. Dama, M. Parrinello, G. A. Voth, Phys Rev Lett 2014, 112, 240602.

[13] F. Aydin, R. Sun, J. M. J. Swanson, Biophys J 2019, 117, 87.

[14] S. Plimpton, Journal of Computational Physics 1995, 117, 1.

[15] G. A. Tribello, M. Bonomi, D. Branduardi, C. Camilloni, G. Bussi, Computer Physics Communications 2014, 185, 604.

[16] W. L. Jorgensen, D. S. Maxwell, J. Tirado-Rives, Journal of the American Chemical Society 1996, 118, 11225.

[17] L. Zhang, J. Yang, X. Wang, B. Zhao, G. Zheng, Nanoscale Res. Lett. 2014, 9.

[18] B. J. Hinds, N. Chopra, T. Rantell, R. Andrews, V. Gavalas, L. G. Bachas, Science 2004, 303, 62.

[19] F. Du, L. T. Qu, Z. H. Xia, L. F. Feng, L. M. Dai, Langmuir 2011, 27, 8437.

[20] Y. Baek, C. Kim, D. K. Seo, T. Kim, J. S. Lee, Y. H. Kim, K. H. Ahn, S. S. Bae, S. C. Lee, J. Lim, K. Lee, J. Yoon, J. Membr. Sci. 2014, 460, 171.

[21] S.-M. Park, J. Jung, S. Lee, Y. Baek, J. Yoon, D. K. Seo, Y. H. Kim, Desalination 2014, 343, 180.

[22] P. Krishnakumar, P. B. Tiwari, S. Staples, T. Luo, Y. Darici, J. He, S. M. Lindsay, Nanotechnol. 2012, 23.

[23] W. L. Mi, Y. S. Lin, Y. D. Li, J. Membr. Sci. 2007, 304, 1.

[24] L. Zhang, B. Zhao, X. Wang, Y. Liang, H. Qiu, G. Zheng, J. Yang, Carbon 2014, 66, 11.

[25] M. Majumder, N. Chopra, R. Andrews, B. J. Hinds, Nature 2005, 438, 44.

[26] M. Majumder, N. Chopra, B. J. Hinds, ACS nano 2011, 5, 3867.

[27] J. Wu, K. Gerstandt, H. Zhang, J. Liu, B. J. Hinds, Nature Nanotechnology 2012, 7, 133.

[28] S. Kim, F. Fornasiero, H. G. Park, J. Bin In, E. Meshot, G. Giraldo, M. Stadermann, M. Fireman, J. Shan, C. P. Grigoropoulos, O. Bakajin, J. Membr. Sci. 2014, 460, 91.

[29] F. Fornasiero, J. Bin In, S. Kim, H. G. Park, Y. Wang, C. P. Grigoropoulos, A. Noy, O. Bakajin, Langmuir 2010, 26, 14848; F. Fornasiero, H. G. Park, J. K. Holt, M. Stadermann, C. P. Grigoropoulos, A. Noy, O. Bakajin, Proceedings of the National Academy of Sciences 2008, 105, 17250.

[30] J. K. Holt, H. G. Park, Y. Wang, M. Stadermann, A. B. Artyukhin, C. P. Grigoropoulos, A. Noy, O. Bakajin, Science 2006, 312, 1034.

[31] Y. Marcus, in *Ions in Solution and their Solvation*, (Ed: Wiley), 2015, 107.

[32] A. K. Geim, K. Gopinadhan, 2017, 513, 511.

[33] S. M. Chathoth, E. Mamontov, S. Dai, X. Wang, P. F. Fulvio, D. J. Wesolowski, Epl-Europhys Lett 2012, 97.

[34] Q. Berrod, F. Ferdeghini, P. Judeinstein, N. Genevaz, R. Ramos, A. Fournier, J. Dijon, J. Ollivier, S. Rols, D. Yu, R. A. Mole, J. Zanotti, Nanoscale 2016, 8, 7845.

[35] C. Pinilla, M. G. D. Po, R. M. Lynden-bell, J. Kohanoff, 2005, 17922.

[36] A. Ghoufi, A. Szymczyk, P. Malfreyt, Scientific reports 2016, 6, 28518.

[37] V. V. Chaban, O. V. Prezhdo, ACS nano 2014, 8, 8190.

[38] R. M. Lynden-Bell, J. C. Rasaiah, The Journal of Chemical Physics 1996, 105, 9266.

[39] J. Kong, Z. Bo, H. Yang, J. Yang, X. Shuai, J. Yan, K. Cen, Physical Chemistry Chemical Physics 2017, 19, 7678.

[40] P. Pohl, S. M. Saparov, Y. N. Antonenko, Biophysical Journal 1998, 75, 1403.

[41] T. Mouterde, L. Bocquet, The European Physical Journal E 2018, 41, 148.

[42] P. Dechadilok, W. M. Deen, 2006, 6953.

[43] C. Peter, G. Hummer, 2005, 89, 2222.

[44] R. K. Joshi, P. Carbone, F. C. Wang, V. G. Kravets, Y. Su, I. V. Grigorieva, H. A. Wu, A. K. Geim, R. R. Nair, Science 2014, 343, 752.

[45] P. R. Kidambi, D. Jang, J. C. Idrobo, M. S. H. Boutilier, L. D. Wang, J. Kong, R. Karnik, Advanced Materials 2017, 29.
